# Targeting Bone Tumor and Subcellular Endoplasmic Reticulum via Near Infrared II Fluorescent Polymer for Photodynamic‐Immunotherapy to Break the Step‐Reduction Delivery Dilemma

**DOI:** 10.1002/advs.202201819

**Published:** 2022-06-26

**Authors:** Xianghong Zhang, Jia Wan, Fuhao Mo, Dongsheng Tang, Haihua Xiao, Zhihong Li, Jinpeng Jia, Tang Liu

**Affiliations:** ^1^ Department of Orthopedics The Second Xiangya Hospital Central South University Changsha Hunan 410011 P. R. China; ^2^ Beijing National Laboratory for Molecular Sciences State Key Laboratory of Polymer Physics and Chemistry Institute of Chemistry Chinese Academy of Sciences Beijing 100190 P. R. China; ^3^ State Key Laboratory of Advanced Design and Manufacture for Vehicle Body Hunan University Changsha Hunan 410082 P. R. China; ^4^ Hunan Key Laboratory of Tumor Models and Individualized Medicine The Second Xiangya Hospital Central South University Changsha Hunan 410011 P. R. China; ^5^ Senior Department of Orthopedics the Fourth Medical Center of PLA General Hospital Beijing 100853 P. R. China

**Keywords:** cascade targeting, endoplasmic reticulum stress, photodynamic‐immunotherapy, step‐reduction delivery dilemma

## Abstract

Specific localization of photosensitizers (PSs) to a certain organelle could result in targeted attack to cause greater trauma to cancer cells, eventually maximizing photodynamic therapy (PDT). However, currently, efficient and precise transportation of PSs via drug delivery to tumor cells and subcellular organelles is still challenging, due to a so‐called step‐reduction delivery dilemma (SRDD) which also threatens anticancer drug delivery to exert their efficacy. Herein, a cascade targeting near infrared II (NIR II) fluorescent nanoparticles (NP^ER/BO‐PDT^) is designed that can target bone tumor first and then target the subcellular organelle of endoplasmic reticulum (ER). It is found that NP^ER/BO‐PDT^ achieves the targeted accumulation of the bone tumor and then ER. NP^ER/BO‐PDT^ generates reactive oxygen species (ROS) in the subcellular organelles of ER under near infrared light irradiation. The continuous ER stress by ROS promotes the release of more damage‐associated molecular patterns, induces immunogenic cell death, stimulates the adaptive immune response, and further synergistically inhibits tumor growth, achieving the so‐called photodynamic‐immunotherapy. Overall, this study exemplifies a safe and efficient nano‐drug delivery system for a bone and ER cascade targeting via delivery of PSs to break the SRDD and highlights potential clinical translation.

## Graphical abstract

To break the step‐reduction delivery dilemma (SRDD), near infrared II fluorescent cascade targeting nanoparticle (NP^ER/BO‐PDT^) is successfully prepared. NP^ER/BO‐PDT^ has the endoplasmic reticulum (ER) targeting ability, resulting in stimulated continuous reactive oxygen species‐based ER stress and cell death. Furthermore, NP^ER/BO‐PDT^ could better break the SRDD and amplify the effect of photodynamic‐immunotherapy in vitro and in vivo.

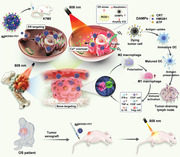

## 1 Introduction

Multiple physiologic physical barriers of solid tumors exist including diseased organs, tissues, cells, and even subcellular organelles which results in the delivery of any drugs to the final targeted following a step‐reduction manner and greatly threatens anticancer drugs to exert their efficacy. This phenomenon greatly hinders the limited progress and clinical translation of nanomedicine. To some extent, it could be actually considered as a step‐reduction delivery dilemma (SRDD).^[^ ^1^ ^]^ It seems imperative to propose new strategies to break this SRDD to improve current cancer therapy.

Photodynamic therapy (PDT) is a promising anti‐tumor therapy with high effectiveness and low incidence of adverse effects.^[^ ^2^, ^3^, ^4^ ^]^ Recent studies unveil PDT can efficiently kill tumor cells through photodynamic‐immunotherapy.^[^ ^3^, ^4^, ^5^ ^]^ However, there is still SRDD which hampered the efficient delivery of photosensitizers (PSs) to exert PDT. Therefore, it is of great significance to deliver PSs to cancer cells and even organelles before they are irradiated.

Osteosarcoma (OS) is a malignant tumor that originates from bone and destroys healthy bone tissues.^[^ ^6^ ^]^ The complex anatomical characteristics and abnormal cancer cell proliferation make the limited vascular systems more unevenly distributed in OS, resulting in the fact that few anticancer drugs can reach the bone tumor tissues.^[^ ^7^ ^]^ Therefore, high‐doses of anticancer agents were used to achieve desirable therapeutic outcomes, which in turn inevitably results in adverse cytotoxic effects and significantly narrows the treatment options for OS.^[^ ^8^ ^]^ Fortunately, the high concentration of minerals in bone tissues is unique for targeted drug delivery.^[^ ^9^ ^]^ Bisphosphonates are such a class of bone targeting agents that can chelate with Ca^2+^ of hydroxyapatite and adhere to the bone tissues.^[^ ^10^ ^]^ Endoplasmic reticulum (ER) is a cell organelle for protein synthesis which determines the function, fate, and survival of cells.^[^ ^11^ ^]^ Moreover, ER stress determines a variety of tumor‐promoting properties, making targeting subcellular organelle ER promising.^[^ ^12^, ^13^, ^14^ ^]^ Recent advances in biomaterials and biosafety materials provide with new possibilities to overcome these limitations.^[^ ^15^ ^]^ Therefore, ideally, PSs could be delivered to the tumor cells and then translocated to ER for photodynamic‐immunotherapy.

**Scheme 1:**
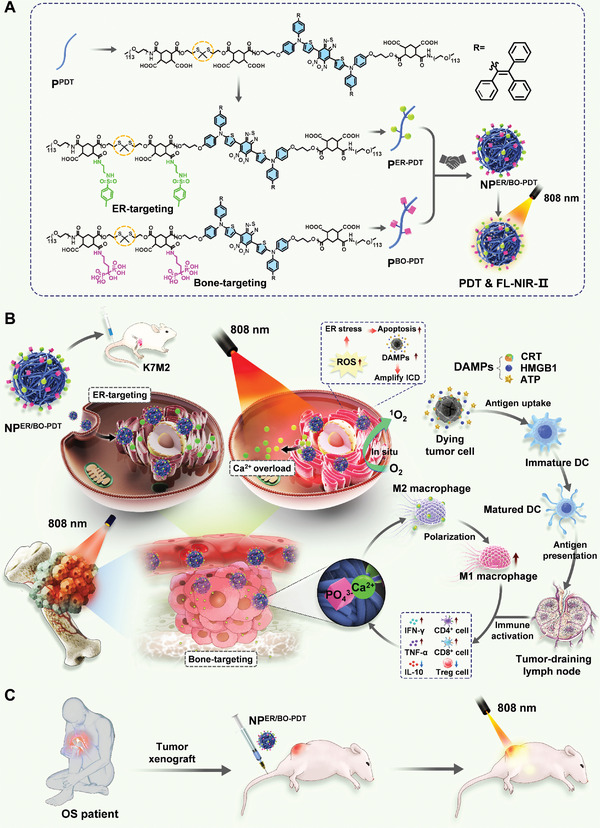
Schematic illustration of NIR II fluorescent NP^ER/BO‐PDT^ with cascade targeting performance for breaking SRDD to amplify photodynamic‐immunotherapy. A) The ER targeting N‐Tosylethylenediamine and bone targeting alendronic acid were respectively introduced into polymer P^PDT^ (ROS generation) to prepare a new ER‐targeting polymer (P^ER‐PDT^) and a bone targeting polymer (P^BO‐PDT^), respectively. Finally, P^ER‐PDT^ and P^BO‐PDT^ were mixed and co‐assemble to NP^ER/BO‐PDT^. B) Inhibition of tumor growth via NP^ER/BO‐PDT^ on a K7M2 cancer model of OS. NP^ER/BO‐PDT^ was injected into mice bearing orthotopic K7M2 OS, and they were accumulated in bone tumor tissues via bone targeting. Subsequently, NP^ER/BO‐PDT^ within the tumor cells could be further delivered to the ER lumen. Upon near infrared (NIR) light irradiation, NP^ER/BO‐PDT^ could generate ROS in ER, which then killed tumor cells directly and triggered the imbalance of Ca^2+^ homeostasis. The persistent imbalance of intracellular Ca^2+^ homeostasis could induce the continuous ROS‐based ER stress, which then increased DAMPs releasing by dying tumor cells, and amplified the ICD effect and activated the adaptive immunity. C) Inhibition of tumor growth via NP^ER/BO‐PDT^ on a second cancer model of PDX^OS^.

Herein, we designed a polymer containing an aggregation‐induced emission (AIE) molecular unit in the main chain, which can produce reactive oxygen species (ROS) and emit near infrared II (NIR II) fluorescence (designated as P^PDT^) for photodynamic‐immunotherapy. Subsequently, an ER‐targeting ligand (N‐Tosylethylenediamine) and a bone targeting ligand (alendronic acid) were introduced into P^PDT^ respectively to prepare an ER‐targeting polymer (designated as P^ER‐PDT^) and a bone targeting polymer (designated as P^BO‐PDT^). Thereafter, P^ER‐PDT^ and P^BO‐PDT^ were mixed and self‐assembled into NIR II fluorescent cascade targeting nanoparticle (NP^ER/BO‐PDT^) (**Scheme** **1**A). NP^ER/BO‐PDT^ with cascade targeting bone tumor and ER performance could break the SRDD. To address this, NP^ER/BO‐PDT^ were injected into mice bearing orthotopic OS and they reached the tumor site through blood circulation. The targeted accumulation of NP^ER/BO‐PDT^ in bone tumor tissues is achieved by alendronic acid mediated chelation with the Ca^2+^ of hydroxyapatite at the bone tumor site, and then the NP^ER/BO‐PDT^ were effectively endocytosed by tumor cells. Finally, NP^ER/BO‐PDT^ were selectively accumulated on the subcellular organelles of ER. Under 808 nm light irradiation, on the one hand, NP^ER/BO‐PDT^ can generate ROS in ER for killing tumor cells; on the other hand, the ROS generated in situ destroys the calcium homeostasis, triggers stronger ER stress, increases damage‐associated molecular patterns (DAMPs) released by dying tumor cells, and amplifies the immunogenic cell death (ICD) effect. Moreover, under the stimulation of DAMPs, immature dendritic cells (DCs) become mature, thereby enhancing their antigen presentation ability and stimulating the effective adaptive immune response. Meanwhile, NP^ER/BO‐PDT^ can promote the M1 polarization of tumor‐associated macrophages (TAMs) and reduce the percentage of regulatory T cells (Tregs) in the tumor microenvironment, and further synergistically activate T cells and regulate the secretion of inflammatory factors, and ultimately achieve efficient photodynamic‐immunotherapy (Scheme 1B). We further established a patient‐derived OS animal model (PDX^OS^), and demonstrated NP^ER/BO‐PDT^ mediated PDT significantly inhibited the tumor growth on a PDX^OS^ model (Scheme 1C). Overall, we demonstrated here NP^ER/BO‐PDT^ target bone and ER for ROS generation, resulting in induction of persistent ER stress, amplifying the ICD effect, which breaks the SRDD and finally highlights the use of cascade targeting strategy for more effective photodynamic‐immunotherapy.

## 2 Results and Discussion

### 2.1 Preparation and Characterization of NP^ER‐PDT^ and NP^ER/BO‐PDT^

P^PDT^ was synthesized via a condensation polymerization as previously described (Scheme S1 and Figure S1, Supporting Information).^[^ ^4^ ^]^ Next, an ER targeting ligand, that is, the N‐Tosylethylenediamine was introduced into P^PDT^ to prepare an ER‐targeting polymer (P^ER‐PDT^) (Scheme S2 and Figure S2, Supporting Information). Similarly, alendronic acid was introduced into P^PDT^ to prepare a bone targeting polymer (P^BO‐PDT^) (Scheme S3, Figures S3 and S4, Supporting Information). Subsequently, P^PDT^ and P^ER‐PDT^ were used to prepare NP^PDT^ and NP^ER‐PDT^, respectively. Moreover, P^ER‐PDT^ and P^BO‐PDT^ were mixed to prepare cascade targeting nanoparticles NP^ER/BO‐PDT^.

**Figure 1:**
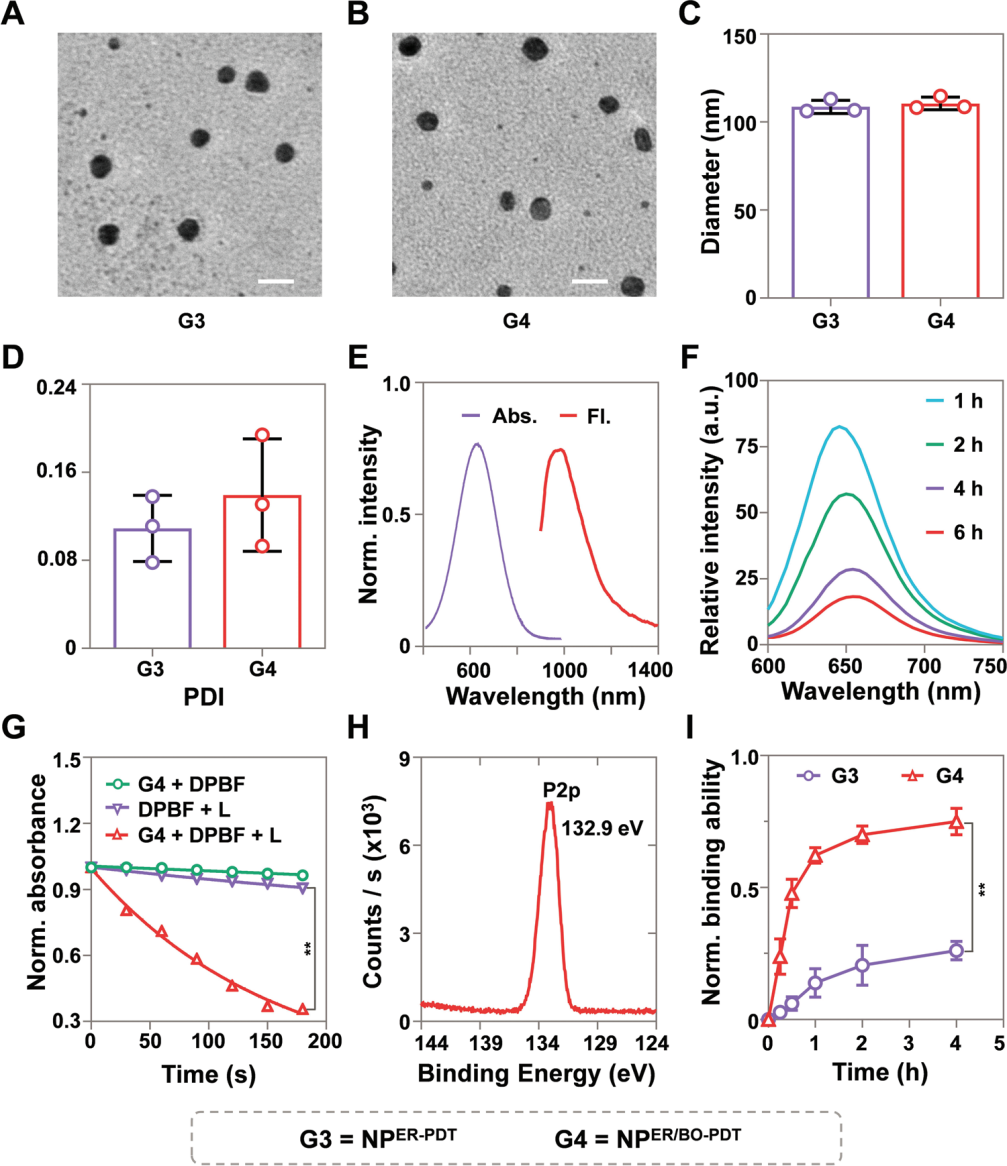
Characterization of NP^ER/BO‐PDT^. A,B) The transmission electron microscope images of NP^ER‐PDT^ and NP^ER/BO‐PDT^. Scale bar: 200 nm. C) The diameter and D) PDI of NP^ER‐PDT^ and NP^ER/BO‐PDT^ by dynamic light scattering. E) Absorption spectra and fluorescence emission spectra of NP^ER/BO‐PDT^. F) NP^ER/BO‐PDT^ dissociation kinetics monitored by Nile red assay. G) Decomposition rates of DPBF by ROS, generated from NP^ER/BO‐PDT^ under NIR light irradiation (808 nm, 1.0 W cm^–2^). H) Characterization of P^BO‐PDT^ by XPS. I) The normalized hydroxyapatite‐binding capacity of NP^ER/BO‐PDT^. Data are shown as mean ± SD. **p < 0.01.

First, we found NP^ER‐PDT^ and NP^ER/BO‐PDT^ exhibited uniform spherical shapes with a homogenous diameter around 100 nm (**Figure** **1**A,B). The average hydrodynamic diameter of NP^ER‐PDT^ and NP^ER/BO‐PDT^ were further confirmed by dynamic light scattering to be 109 and 112 nm with polydispersity indexes (PDI) at 0.11 and 0.14, respectively (Figure 1C,D). To further verify the photostability of NP^ER/BO‐PDT^, we then irradiate NP^ER/BO‐PDT^ under 808 nm laser for 10 min with Indocyanine Green (ICG) as a control. Results showed that the color of NP^ER/BO‐PDT^ was basically unchanged, while ICG has an obvious photobleaching effect, indicating NP^ER/BO‐PDT^ had a better light stability (Figure S5A, Supporting Information). Moreover, the diameter of NP^ER/BO‐PDT^ did not change after 10 days in PBS, further proving the good stability of NP^ER/BO‐PDT^ (Figure S5B, Supporting Information). Next, we study the absorbance and light emitting properties of nanoparticles. The results showed that NP^ER/BO‐PDT^ had a strong absorption in the range of 300–850 nm with a maximum absorption peak at 688 nm. Moreover, under 808 nm light irradiation, NP^ER/BO‐PDT^ demonstrated strong fluorescence emission in the second NIR window (NIR II, 950–1700 nm) with a major peak at 1001 nm (Figure 1E). In addition, NP^ER/BO‐PDT^ was supposed to be ROS sensitive as there are numerous thioketal bonds and AIE molecular units in the polymer main chain. Under NIR light irradiation, NP^ER/BO‐PDT^ can generate ROS, thereby breaking down the thioketal bonds, resulting in the dissociation of nanoparticles. To verify this process, on the one hand, H_2_O_2_ was used to dissociate the NP^ER/BO‐PDT^ with Nile red, and the changes in the absorption peak of Nile red were recorded for the nanoparticle dissociation kinetics.^[^ ^16^ ^]^ The results showed that the dissociation half‐life of NP^ER/BO‐PDT^ is ≈2.71 h (Figure 1F). On the other hand, since robust ROS generation was a prerequisite for PDT, we continued to study the ROS generation ability of NP^ER/BO‐PDT^ under NIR light irradiation (808 nm, 1.0 W cm^–2^). DPBF (1,3‐Diphenylisobenzofurane) is selected as a ROS indicator because it would be oxidized and degraded by ROS, making the absorbance of DPBF at 415 nm ideal for monitoring the level of ROS generation. Results showed that under NIR light irradiation for 180 s, the absorbance of NP^ER/BO‐PDT^ and DPBF aqueous mixture solution significantly reduced with a half‐life of 110 s, and only 35.6% of DPBF is not oxidized (Figure 1G), indicating that NP^ER/BO‐PDT^ can quickly generate ROS (the singlet oxygen, ^1^O_2_) under NIR light irradiation. Finally, to demonstrate the bone targeting ability, on the one hand, P^BO‐PDT^ with phosphate was characterized by XPS with obvious peaks at 132.9 eV (Figure 1H), indicating P^BO‐PDT^ was successfully conjugated with alendronic acid. On the other hand, the binding ability of NP^ER/BO‐PDT^ to hydroxyapatite was studied and we found that NP^ER/BO‐PDT^ can bind to hydroxyapatite faster, and the binding force of NP^ER/BO‐PDT^ to hydroxyapatite is about three times that of NP^ER‐PDT^ (Figure 1I). The above results together indicated that NP^ER/BO‐PDT^ had bone targeting ability.

### 2.2 ER‐Targeting and Cytotoxicity of NP^ER/BO‐PDT^

**Figure 2:**
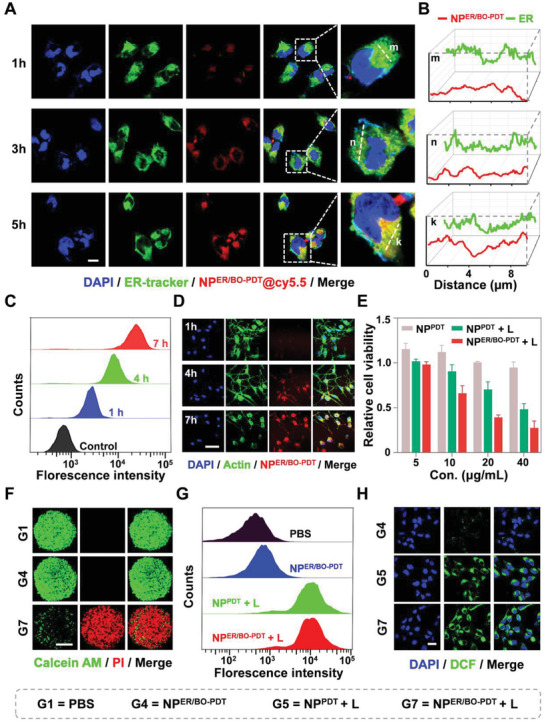
NP^ER/BO‐PDT^ targets ER to generate ROS in situ. A) ER targeting of NP^ER/BO‐PDT^ in MNNG/HOS cells. ER was stained with ER Tracker (green). MNNG/HOS cells were incubated with NP^ER/BO‐PDT^@Cy5.5 for different durations, Scale bar: 10 µm. B) The fluorescence intensity at lines m, n, and k in (A), the red and green curves represent the fluorescence intensity from NP^ER/BO‐PDT^@Cy5.5 and ER (Green), respectively. C) Quantitative study of the intracellular uptake of NP^ER/BO‐PDT^@Cy5.5 by FCM at different durations. D) Intracellular uptake of NP^ER/BO‐PDT^ (red) by K7M2 cells by CLSM (NIR II, 808 nm), and the cell skeleton was stained by Actin (green), Scale bar: 50 µm. E) Cell viability of K7M2 cells with various treatments by MTT. F) Live‐dead assay on 3D tumor spheroids of K7M2 cells with various treatments. The living cells are presented as green by Calcein‐AM, and the dead cells are present as red by PI. Scale bar: 50 µm. G) Intracellular ROS generation with various treatments by FCM. H) CLSM images of intracellular ROS level of MNNG/HOS cells. The green came from DCF generated via the reaction of DCFH‐DA and ROS. Scale bar: 20 µm. Data are shown as mean ± SD.

The uptake of NP^ER/BO‐PDT^ by tumor cells and further selective accumulation in the ER play a critical role in its anti‐tumor activity. On the one hand, the ER‐targeting property of NP^ER/BO‐PDT^ was examined in MNNG/HOS cells by labeling NP^ER/BO‐PDT^ with Cy5.5 dye (NP^ER/BO‐PDT^@Cy5.5). As shown in **Figure** **2**A, the co‐location rate exhibited a time‐dependent cellular uptake, which gradually enhanced as the incubation time increased. Upon incubation for 5 h, the red fluorescence signal from NP^ER/BO‐PDT^@Cy5.5 and the green signal from ER tracker matched well (the Pearson correlation coefficient is 0.61), making yellow fluorescent spots within ER, which demonstrated the specific ER targeting ability of NP^ER/BO‐PDT^. Subsequently, the red and green fluorescence intensity at lines m, n, and k in Figure 2A was further analyzed. The line‐scan profiles also denoted the co‐localization of NP^ER/BO‐PDT^ within ER compartments (Figure 2B). These results verified that NP^ER/BO‐PDT^ possessed selectivity for ER in cells. On the other hand, NP^ER/BO‐PDT^@Cy5.5 was used to treat the cells for intracellular uptake study and we found the fluorescence intensity in MNNG/HOS cells was gradually intensified with increasing incubation time from 1 h to 7 h by flow cytometry (FCM). Specifically, the fluorescence intensity at 7 h was nearly ten fold higher than that at 1 h (Figure 2C). Fluorescence with high spatial and temporal characteristics can help one understand the physiological function of nanoparticles.^[^ ^17^ ^]^ Here, we could observe NP^ER/BO‐PDT^ via NIR II imaging for cellular endocytosis. Results showed similar trends that the red fluorescence was gradually intensified with increasing incubation time of NP^ER/BO‐PDT^ in K7M2 cells (Figure 2D). 3D multi‐cellular tumor spheroids can better mimic the in vivo cell microenvironment, and narrow the gap between in vivo and in vitro experiments. Subsequently, we further verified the uptake of NP^ER/BO‐PDT^@Cy5.5 with 3D multi‐cellular tumor spheroids. We found the red fluorescence intensity in the same depth portion of 3D tumor spheres was also gradually intensified from 1 h to 7 h (Figure S6A, Supporting Information), hence confirming the effective uptake of NP^ER/BO‐PDT^ by 3D multi‐cellular tumor spheroids. In addition, NP^ER/BO‐PDT^ may enter tumor cells through endocytosis. To prove this process, we treated 3D multi‐cellular tumor spheroids with an endocytosis inhibitor genistein. Results unveiled that genistein can inhibit the endocytosis of NP^ER/BO‐PDT^ (Figure S6B, Supporting Information), indicating NP^ER/BO‐PDT^ can indeed be effectively endocytosed by tumor cells.

After tumor cells take up NP^ER/BO‐PDT^, it will generate ROS under NIR light irradiation, and then kills tumor cells. First, the anti‐tumor activity of NP^ER/BO‐PDT^ was evaluated in vitro. As shown in Figure 2E, NP^ER/BO‐PDT^ + L had a 62% inhibition rate at 20 µg mL^–1^ in K7M2 cells, while NP^PDT^ + L only had a 30% inhibition rate at the same concentration. Similar results were found in other OS cells (Figure S7A,B, Supporting Information). Moreover, the apoptosis rate of cells treated with NP^ER/BO‐PDT^ revealed that NP^ER/BO‐PDT^ + L induced significantly higher level of apoptosis (47.6%) as compared to NP^PDT^ + L (26.9%) in K7M2 cells (Figure S8A,B, Supporting Information). Taken together, these results indicated that NP^ER/BO‐PDT^ with ER‐targeting ability had stronger anti‐tumor activity and pro‐apoptotic effect than NP^PDT^. Second, live and dead assay was applied to investigate the cell killing effect of NP^ER/BO‐PDT^ in 2D and 3D tumor spheroids. The results revealed that the tumor cells and spheroids treated with PBS or NP^ER/BO‐PDT^ displayed mainly green fluorescence (live cells), while those treated with NP^ER/BO‐PDT^ + L showed great red fluorescence (dead cells) (Figure S9, Supporting Information and Figure 2F). Third, we continued to evaluate the ROS generation ability of NP^ER/BO‐PDT^ via DCFH‐DA probes by FCM and confocal laser scanning microscopy (CLSM). We found that on the one hand, the fluorescence intensity of cells treated with NP^ER/BO‐PDT^ + L was nearly ten fold higher than that of cells treated with NP^ER/BO‐PDT^ without laser irradiation by FCM (Figure 2G and Figure S10A, Supporting Information). On the other hand, cells treated with NP^PDT^ + L or NP^ER/BO‐PDT^ + L all exhibited strong green fluorescence by CLSM, further proving the efficient ROS generation (Figure 2H). As the half‐life of ROS in cells is very short (< 40 ns) and the intracellular diffusion distance is limited (<20 nm), the ROS generated in situ in subcellular organelles prompts us to design cascade targeting nanoparticles for enhancing the anti‐tumor efficacy.^[^ ^18^ ^]^

### 2.3 NP^ER/BO‐PDT^ Induces the ER Stress and ICD

**Figure 3:**
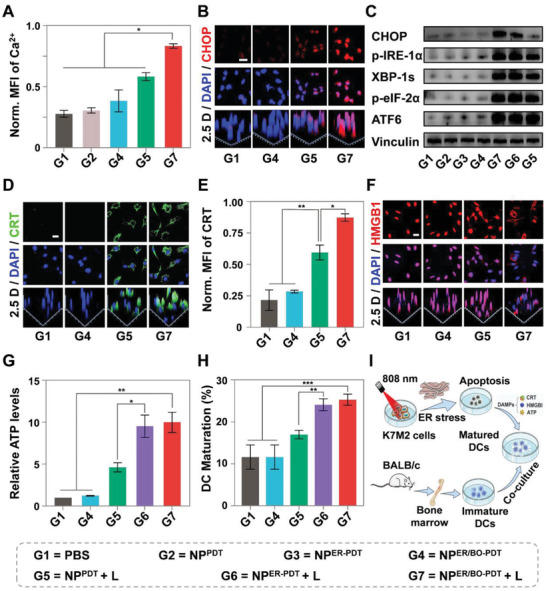
NP^ER/BO‐PDT^ induced the imbalance of Ca^2+^ in the ER lumen and stimulated persistent ROS‐based ER stress, resulting in a stronger ICD effect and more DCs maturation under NIR light irritation in vitro. A) The cytosolic Ca^2+^ level in cells after various treatments by FCM. B) CLSM images of CHOP‐stained cells after various treatments. Scale bar: 20 µm. C) Western blot results of the ER stress proteins after various treatments. D) CLSM images of the exposure of CRT in K7M2 cells after various treatments. Scale bar: 20 µm. E) Quantitative study of the exposure of CRT by FCM. F) CLSM images of the release of HMGB1 in K7M2 cells after various treatments. Scale bar: 20 µm. G) The relative ATP release of cells after various treatments. H) Quantitative study of the maturation of BMDCs co‐cultured with K7M2 cells with various pretreatments by FCM. I) Schematic illustration of ER‐targeting of NP^ER/BO‐PDT^ for PDT, resulting in the ICD effect and further maturation of BMDCs in vitro. Data are shown as mean ± SD. Student's *t*‐test. *p < 0.05, **p < 0.01, ***p < 0.001.

ER is a complex dynamic organelle with an important intracellular Ca^2+^ store.^[^ ^19^ ^]^ Generally, intracellular ROS levels are always maintained in a state of dynamic equilibrium. Once there is too much intracellular ROS generated, the excessive ROS will impair the ER function, which subsequently results in the imbalance of intracellular Ca^2+^ homeostasis.^[^ ^20^ ^]^ To confirm whether the NP^ER/BO‐PDT^ would induce more dramatic imbalance of Ca^2+^ homeostasis under continuous NIR light irradiation, the intracellular Ca^2+^ fluorescence intensity was detected by CLSM and FCM. A Ca^2+^ probe Fluo‐3 AM was employed which could be cleaved in cancer cells by enzymes to form Fluo‐3. Once the Fluo‐3 meets with Ca^2+^, there is green fluorescence. We showed that cells treated with NP^ER/BO‐PDT^ + L exhibited stronger green fluorescence by CLSM, which was 1.9 times higher than that of NP^PDT^ + L (Figure S10B,C, Supporting Information). The further quantification analysis via FCM revealed the fluorescence intensity of cells treated with NP^ER/BO‐PDT^ + L was 1.6 times higher than that of NP^PDT^ + L (**Figure** **3**A). Taken together, these results proved NP^ER/BO‐PDT^ can dramatically impair the stability of Ca^2+^ homeostasis than NP^PDT^ under NIR light irradiation.

The persistent imbalance of intracellular Ca^2+^ homeostasis would impair the proper synthesis and folding of proteins, which consequently induces the accumulation of unfolded or misfolded proteins in the ER lumen, accompanied by ER stress.^[^ ^20^ ^]^ Moreover, the degree of ER stress determines whether to return homeostasis or to activate the cell death program. At the initiation of ER stress, misfolded proteins inflicted by the excessive ROS would bind to the ER chaperone binding immunoglobin protein (BiP), which then leaves the ER stress sensors protein kinase‐like endoplasmic reticulum kinase (PERK), activating transcription factor‐6 (ATF6), and inositol‐requiring enzyme 1*α* (IRE1*α*) free to be activated. Additionally, these activated ER stress responsive proteins would further upregulate the unfolded protein response (UPR), which facilitates the clearance of unfolded or misfolded proteins and the maintenance of ER homeostasis. However, once the status of ER stress continues for a long time, the expression of proapoptotic C/EPB homologous protein (CHOP), which is a key mediator of the ER stress‐mediated apoptosis pathway, would be upregulated by the excess ROS through PERK/eIF2*α*/ATF6/CHOP pathway, and thereby promoting cell apoptosis.^[^ ^12^, ^21^ ^]^ To briefly prove the above mentioned ER stress process in MNNG/HOS cells under various treatments, we carried out a relative protein expression analysis via CLSM and Western blot. CLSM imaging revealed that the red fluorescence signal from CHOP and the blue signal from cell nucleus stained with DAPI showed pink fluorescence in the merged images in cells treated with NP^ER/BO‐PDT^ + L, demonstrating the significant upregulated expression of CHOP (Figure 3B). Furthermore, the Western blot results revealed that the expression of CHOP, p‐IRE‐1*α*, p‐eIF2*α*, ATF6, and XPB‐1s were significantly upregulated in cells treated with NP^ER/BO‐PDT^ + L, as compared to those treated with NP^PDT^ + L, especially for CHOP (Figure 3C). Taken together, the aforementioned results fully validated that NP^ER/BO‐PDT^ + L was capable of inducing a considerable ROS generation in ER, which then contributed to the redox balance disorder and the imbalance of Ca^2+^ homeostasis, and stimulated continuous ROS‐based ER stress and cell death through activation of the PERK/eIF2*α*/ATF6/CHOP pathway.

Recently, more and more studies have demonstrated that PDT can induce ICD effect through the ROS‐based ER stress effect.^[^ ^5^, ^22^ ^]^ Tumor cells that undergo ICD would generate a series of DAMPs, such as surface‐exposed calreticulin (CRT) from the ER lumen, passively released high mobility group Box 1 (HMGB1), and secreted adenosine triphosphate (ATP) from the cytoplasm, which are also the hallmarks of ICD.^[^ ^14^, ^22^, ^23^ ^]^ Specifically, the surface‐exposed CRT is a prophagocytic “eat me” signal, and the secreted ATP can serve as a “find‐me” signal that jointly elicits phagocytosis of the dying tumor cells by the DCs.^[^ ^24^ ^]^ HMGB1 can promote DCs maturation and antigen presentation.^[^ ^25^ ^]^ Taken together, all these DAMPs coopted in triggering enhanced immune responses.^[^ ^26^ ^]^ Hence, to verify whether the ROS‐based ER stress triggered by NP^ER/BO‐PDT^ can induce effective ICD, promote the release of DAMPs, and induce the DCs to mature under NIR light irradiation, we conducted a series of experiments. First, the surface‐exposed CRT was detected by CLSM and FCM. On the one hand, the result of CLSM study showed that K7M2 cells treated with NP^ER/BO‐PDT^ + L exhibited stronger green fluorescence than those treated with NP^PDT^ + L, indicating higher exposure of CRT (Figure 3D). The quantification of the fluorescence pixel intensity in each cell further revealed that the green fluorescence in cells treated with NP^ER/BO‐PDT^ + L was ≈1.4 times higher than that of NP^PDT^ + L (Figure S11A, Supporting Information). On the other hand, the quantitative FCM result further suggested that the fluorescence intensity of CRT in cells treated with NP^ER/BO‐PDT^ + L was 1.5 times higher than that of NP^PDT^ + L (Figure 3E). The aforementioned results prove that NP^ER/BO‐PDT^ triggered higher CRT expression under NIR light irradiation. Second, we continued to investigate the translocation of HMGB1 from nucleus to extracellular matrix. As shown in Figure 3F, the HMGB1 (red) was primarily merged with the nucleus (blue) of K7M2 cells treated with PBS. However, compared with NP^PDT^ + L, more HMGB1 was released from the nucleus in the cells treated with NP^ER/BO‐PDT^ + L (Figure S11B, Supporting Information). Taken together, the above findings indicated that NP^ER/BO‐PDT^ promotes more translocation of HMGB1 under NIR light irradiation. Third, ATP secretion in the cell culture medium was evaluated by ATP assay. As shown in Figure 3G, ATP secretion in the supernatant of NP^ER/BO‐PDT^ + L treated cells was almost 2.2 times than that of NP^PDT^ + L, indicating that cell treated with NP^ER/BO‐PDT^ promoted the higher level of ATP secretion under NIR light irradiation. Finally, to confirm whether the ICD effect induced by DAMPs can promote the maturation of bone marrow‐derived dendritic cells (BMDCs), we continued to evaluate the DCs maturation in vitro through co‐incubation with K7M2 cells following various treatments. The results showed that the maturation ratio of co‐cultured DCs treated with NP^ER/BO‐PDT^ + L (25.3%) was 2.2 times higher than that of the PBS treatment group (11.6%), while the ratio in NP^PDT^ + L treatment group was 17.0%, which substantially demonstrated the stronger DCs mature effect of NP^ER/BO‐PDT^ + L (Figure 3H and Figure S12, Supporting Information). In summary, the persistent ROS‐based ER stress triggered by NP^ER/BO‐PDT^ under NIR light irradiation can effectively induce the ICD effect of tumor cells and further promote DCs maturation (Figure 3I).

### 2.4 Biodistribution and Tumor Suppression of NP^ER/BO‐PDT^ in vivo

Biosafety is a prerequisite for nanomedicine to achieve anti‐tumor ability in vivo. Hence, we conducted a safety assessment of NP^ER/BO‐PDT^ first. Healthy KM mice were used and intravenously injected with a single dose of NP^ER/BO‐PDT^. The body weight of mice in each group was recorded every 2 days from the day of injection. Fourteen days after the administration, the blood and main organs of mice were taken for physiological and biochemical examination. On the one hand, the results of blood count and biochemical parameters showed that mice treated with NP^ER/BO‐PDT^ were relatively normal as compared to the PBS group (Figures S13 and S14, Supporting Information). Compared with the PBS group, there was no significant difference in the average body weight of the mice in the NP^ER/BO‐PDT^ treatment group (Figure S15A, Supporting Information). On the other hand, no obvious pathological abnormality was found in the hematoxylin and eosin (H&E) staining images of main organs in the NP^ER/BO‐PDT^ treatment group (Figure S15B, Supporting Information). Altogether, the aforementioned results proved the excellent biosafety of NP^ER/BO‐PDT^, which is important for in vivo applications.

**Figure 4:**
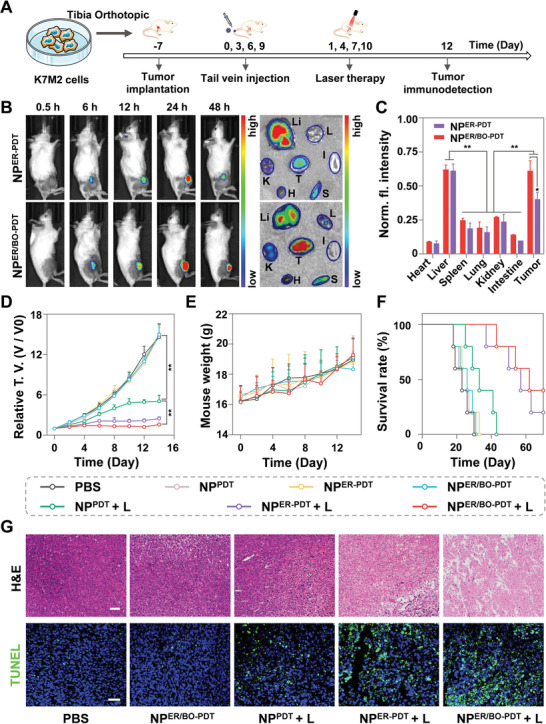
Biodistribution and antitumor effect of NP^ER/BO‐PDT^ on an orthotopic K7M2 tumor model. A) Establishment of an orthotopic K7M2 tumor model for in vivo therapeutic and imaging studies. B) In vivo and ex vivo biodistribution of NP^ER/BO‐PDT^ in K7M2 tumor bearing mice (left panel) and major organs (right panel) via fluorescence bio‐imaging. T, H, Li, S, L, K represent tumor, heart, liver, spleen, lung, and kidney, respectively. C) Semiquantitative biodistribution of NP^ER/BO‐PDT^ in the dissected tumors and major organs by fluorescence quantification at 48 h after intravenous injection. D) Tumor growth curves in mice with various treatments. E) The body‐weight variation and F) survival curve analysis of mice with various treatments. G) H&E staining (upper) and TUNEL staining (lower) in tumors extracted from mice with various treatments. Scale bar: 50 µm. Data are shown as mean ± SD. *p < 0.05, **p < 0.01.

Subsequently, we explored the in vivo biodistribution of NP^ER/BO‐PDT^ on an orthotopic K7M2 OS model based on BALB/c mice (**Figure** **4**A).^[^ ^27^ ^]^ The in vivo imaging showed that the fluorescence intensity of tumor site continued to increase from 0.5 h to 24 h after an intravenous injection by an in vivo Imaging System (IVIS, PerkinElmer). Moreover, the fluorescence intensity of tumor site in NP^ER/BO‐PDT^ treatment group was higher than that of the NP^ER‐PDT^ treatment group at 24 and 48 h after an intravenous injection (Figure 4B, left panel). The above results indicated that NP^ER/BO‐PDT^ had excellent stability and could achieve rapid accumulation and retention in the bone tumor tissues compared to NP^ER‐PDT^. Then, the main organs and tumors of mice at 48 h after injection were further collected and analyzed quantitatively ex vivo. We found that the ex vivo accumulation of NP^ER/BO‐PDT^ in tumor tissues is greater than that of NP^ER‐PDT^, and the fluorescence intensity of NP^ER/BO‐PDT^ in the tumor tissues is ≈1.6 times that of NP^ER‐PDT^ (Figure 4B (right panel) and 4C), further confirming the superior bone targeting ability of NP^ER/BO‐PDT^.

To further evaluate the in vivo anti‐tumor effect of NP^ER/BO‐PDT^, we constructed an orthotopic K7M2 OS model based on BALB/c mice. As shown in Figure 4A, NP^ER/BO‐PDT^ was intravenously injected, and the tumor volume and body weight in each group were recorded every 2 days from the day of treatment began. First, the results showed that the tumor growth inhibition rate of NP^ER/BO‐PDT^ + L (95.3%) was 1.4 times that of NP^PDT^ + L (70.5%), indicating that NP^ER/BO‐PDT^ + L more successfully inhibited the tumor growth (Figure 4D and Figure S16A–H, Supporting Information). Second, the average body weight of the mice treated with NP^ER/BO‐PDT^ + L at day 14 was ≈1.2 times the initial body weight (Figure 4E). Third, the survival rate of mice treated with NP^ER/BO‐PDT^ + L reached 40% after 65 days, while the mice treated with NP^PDT^ + L all died (Figure 4F). Taken together, the above results revealed that NP^ER/BO‐PDT^ + L had better anti‐tumor activity and prolonged survival rate. Finally, the H&E staining and terminal deoxynucleotidyl transferase‐mediated dUTP‐biotin nick end labeling (TUNEL) staining were applied to evaluate the necrosis and apoptotic death in tumor tissues. H&E staining showed that NP^ER/BO‐PDT^ + L induced a larger range of nuclear fragmentation and nuclear lysis in tumors compared to NP^PDT^ + L (Figure 4G, upper). Further TUNEL staining of the tumor slices showed a larger number of apoptotic cells in the NP^ER/BO‐PDT^ + L treatment group (Figure 4G, lower). In summary, compared with NP^PDT^, NP^ER/BO‐PDT^ had higher therapeutic efficacy in vivo under NIR light irradiation.

### 2.5 ICD Induction and Immune Response of NP^ER/BO‐PDT^ in vivo

**Figure 5:**
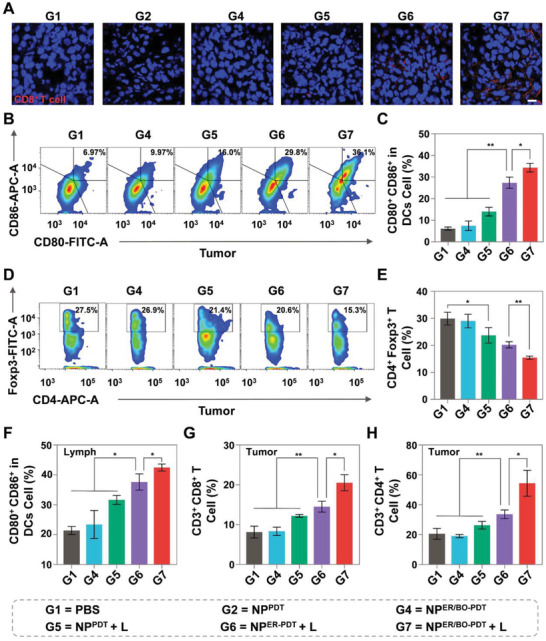
Cascade targeting NP^ER/BO‐PDT^ enhanced the antitumor immunity *in viv* *o*. A) Immunofluorescence staining CD8^+^ positive T cells in mice treated with NP^ER/BO‐PDT^. Scale bar: 10 µm. B) The representative FMC analysis images of DCs and C) the percentages of mature DCs (CD80^+^CD86^+^) populations within tumor tissues. D) The representative FCM analysis images of Tregs (CD4^+^Foxp3^+^) and E) the percentages of Tregs populations with tumor tissues. F) The percentages of mature DCs (CD80^+^CD86^+^) populations within TDLNs. The percentages of G) CD8^+^ T cells populations and H) CD4^+^ T cells populations with tumor tissues. Data are shown as mean ± SD. *p < 0.05, **p < 0.01.

To further explore the ICD effect of NP^ER/BO‐PDT^ and innate immunity in vivo (**Figure** **5**A–H), tumor tissues, spleen, and tumor‐draining lymph nodes (TDLNs) of mice in various treatment groups were harvested for immunofluorescence (IF) and FCM analysis. First, to verify the ER stress, the release of DAMPs, and the infiltration of CD8^+^ T cells, we evaluated the CHOP expression, the CRT exposure, HMGB1 release, and CD8^+^ T cells infiltration (red fluorescence) in tumor tissues after different treatments by IF staining. The results showed that the red fluorescence (CHOP, CRT, and HMGB1) intensity (Figure S17, Supporting Information) in mice treated with NP^ER/BO‐PDT^ + L was higher than other various treatment groups. Similarly, the red fluorescence (CD8^+^ T cells) intensity in NP^ER/BO‐PDT^ + L treatment group was higher than other different treatments (Figure 5A). Moreover, peripheral blood was collected from mice with various treatments on the twelfth day for analyzing cytokine levels. The results showed that the mean levels of TNF‐*α* and IFN‐*γ* in NP^ER/BO‐PDT^ + L treatment group were high than that of NP^PDT^ + L treatment group, while the mean level of IL‐10 in NP^ER/BO‐PDT^ + L treatment group was lower than that of other various treatment groups (Figure S18A–C, Supporting Information). Altogether, under NIR light irradiation, NP^ER/BO‐PDT^ could induce more DAMPs release, more CD8^+^ T cells infiltration, and then effectively induces adaptive immunity in vivo.

The DAMPs signaling molecules released by dying tumor cells can promote the maturation of DCs, and the matured DCs would present antigens to T cells in the lymph nodes, which thereafter effectively stimulates the adaptive immune responses via the activated T lymphocytes.^[^ ^22^ ^]^ First, to explore whether ICD‐induced by NP^ER/BO‐PDT^ + L could contribute to the maturation of DCs in vivo, tumor tissues and TDLNs of mice were harvested for FCM analysis. On the one hand, in tumor tissues, the percentage of matured DCs (CD80^+^ CD86^+^) in mice treated with NP^ER/BO‐PDT^ + L (34.3%) is 1.3 times and 2.5 times that of NP^ER‐PDT^ + L (27.4%) and NP^PDT^ + L (14.0%) (Figure 5B,C and Figure S19A, Supporting Information), respectively. On the other hand, in TDLNs, the percentage of matured DCs (CD80^+^ CD86^+^) in mice treated with NP^ER/BO‐PDT^ + L (42.5%) is 2.0 times that of PBS (21.4%), while the percentage in mice treated with NP^ER‐PDT^ + L (37.6%) and NP^PDT^ + L (31.6%) is 1.8 times and 1.5 times that of PBS (Figure 5F and Figure S20, Supporting Information), respectively. The above results fully illustrated that NP^ER/BO‐PDT^ could dramatically accelerate DCs mature in tumors and TDLNs under NIR light irradiation. Second, to verify whether the matured DCs could evoke the adaptive immune response by activating T lymphocytes, we then evaluated and quantified the infiltration of CD4^+^/CD8^+^ T cells in tumor tissues and spleens via FCM. We found that in tumor tissues the results showed that the percentages of CD4^+^ T cells (54.4%) and CD8^+^ T cells (20.5%) in mice treated with NP^ER/BO‐PDT^ + L are higher than any other treatments, which are 2.6 times and 2.5 times that of the PBS (Figure 5G,H and Figure S21, Supporting Information), respectively. Moreover, the percentage of CD8^+^ T cells by FCM was also consistent with the previous observations with IF staining. Additionally, in the spleens, a more remarkable increase in CD4^+^ T cells and CD8^+^ T cells was observed in mice treated with NP^ER/BO‐PDT^ + L, which showed 1.4 times and 1.8 times that of the PBS (Figures S22A,B and S23, Supporting Information), respectively. The above results have demonstrated ICD effect induced by NP^ER/BO‐PDT^ + L could promote greater antitumor immune response. However, there still exist various immunosuppressive mechanisms protecting tumor cells from being eliminated by the immune system.^[^ ^28^ ^]^ Hence, we further evaluated the M1 polarization of TAMs and the infiltration of the immunosuppressive Tregs in tumor tissues. On the one hand, the results showed that the percentage of M1 polarized macrophages (CD80^+^ CD206^–^) in mice treated with NP^ER/BO‐PDT^ + L was obviously greater than other treatments (Figures S22C and S24, Supporting Information), whereas the changing trend of M2 polarized macrophages (CD80^–^ CD206^+^) was just the opposite (Figure S22D, Supporting Information). The further ratio of M1/M2 in mice treated with NP^ER/BO‐PDT^ + L was 1.5 times of NP^ER‐PDT^ + L and 2.0 times of NP^PDT^ + L (Figure S22E, Supporting Information), respectively, implying NP^ER/BO‐PDT^ + L can promote the M1 polarization of TAMs. On the other hand, as shown in Figure 5D,E, a significant down‐regulation of Tregs was shown in the mice treated with NP^ER/BO‐PDT^ + L. The percentage of Tregs (CD4^+^ FoxP3^+^) in mice treated with NP^PDT^ + L (23.7%) is 1.5 times that of NP^ER/BO‐PDT^ + L (15.4%) (Figure S19B, Supporting Information). Taken together, these results clearly demonstrated that the bone and ER cascade targeting NP^ER/BO‐PDT^ + L effectively induced adaptive immunity in K7M2 tumor‐bearing mice via robust ICD effect, reprogrammed immunosuppressive microenvironment, achieved the synergistic enhancement of PDT and immunotherapy, and provided a new strategy for OS.

**Figure 6:**
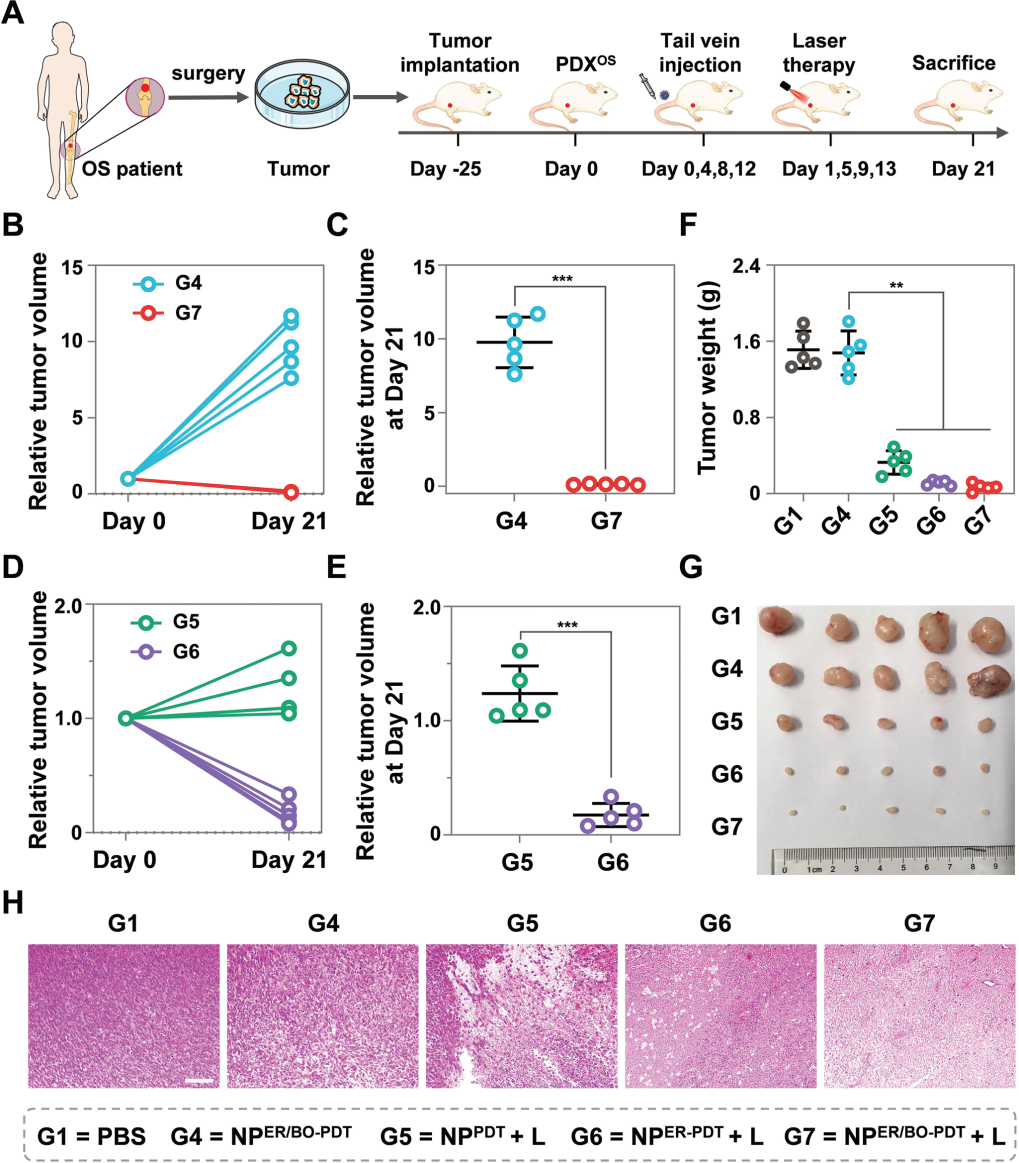
Antitumor effect of NP^ER/BO‐PDT^ on a PDX^OS^ tumor model. A) Schematic illustration of establishment of PDX^OS^ model for in vivo therapeutic study. B–E) Tumor growth profiles of mice with various treatments. F) Tumor weights and G) images after the mice were sacrificed at day 21, and the tumors were isolated. H) Representative photographs of H&E staining images from mice with various treatments. Scale bar: 100 µm. Data are shown as mean ± SD. **p < 0.01, ***p < 0.001.

To further investigate the in vivo antitumor efficacy of NP^ER/BO‐PDT^, we further constructed a PDX^OS^ mice model, which was derived from patients who had not received any chemotherapy or other regimes for OS. When the tumor size reached ≈100 mm^3^, mice bearing PDX^OS^ tumor were randomly divided into five groups. Then, as shown in **Figure** **6**A, NP^ER/BO‐PDT^ was intravenously injected, and the tumor volume and body weight in each group were monitored. The results showed that the tumors in mice of NP^ER/BO‐PDT^ treatment group growing rapidly (Figure S25A–E, Supporting Information), and the average relative tumor volume at day 21 in NP^ER/BO‐PDT^ treatment group was 9.8, while it was 0.13 in NP^ER/BO‐PDT^ + L treatment group (Figure 6B,C). Compared with that of the NP^PDT^ + L treatment group, the tumor growth in NP^ER‐PDT^ + L treatment group was significant inhibited, and the relative tumor volume at day 21 in NP^PDT^ + L treatment group was seven times that of NP^ER‐PDT^ + L treatment group (Figure 6D,E). However, there was no significant difference in the average body weight of the mice in different treatment groups (Figure S25F, Supporting Information). More importantly, tumors in the mice of NP^ER/BO‐PDT^ + L treatment group shrunk drastically, and the tumor weight at day 21 was only 5% of the PBS treatment group (Figure 6F,G). Further H&E staining images of the tumor slices showed a larger number of apoptotic cells in the NP^ER/BO‐PDT^ + L treatment group (Figure 6H). Altogether, under NIR light irradiation, NP^ER/BO‐PDT^ also could induce a significant therapeutic effect on a PDX^OS^ model.

## 3 Conclusions

PDT is a promising cancer treatment modality via photodynamic‐immunotherapy. However, the SRDD is one of the main limiting factors that restrict the anticancer activity. In this study, a cascade targeting NIR II fluorescent nano‐drug delivery system was successfully designed with AIE effects and breaking the SRDD for strong anti‐tumor immunity, which exhibited efficient accumulation and retention at bone tumor site and ER lumen, and excellent therapeutic effect on OS tumor‐bearing mice. We found that NP^ER/BO‐PDT^ could fundamentally improve the effect of photodynamic‐immunotherapy under NIR light irradiation through more efficient accumulation of PSs in tumor tissues and even the subcellular organelles. Specially, potent ICD induced by NP^ER/BO‐PDT^ via persistent and intense ROS‐based ER stress in situ of ER could promote immune recognition, boost strong system immune response, and re‐modulate the immunosuppressive microenvironment. Overall, our finding proposed a promising strategy to enhance photodynamic‐immunotherapy with potential clinical application and transformation prospects.

## Conflict of Interest

The authors declare no conflict of interest.

## Supporting information

Supporting Information

## Data Availability

The data that support the findings of this study are available from the corresponding author upon reasonable request.
