# Supplementary material for: Targeting Bone Tumor and Subcellular Endoplasmic Reticulum via Near Infrared II Fluorescent Polymer for Photodynamic‐Immunotherapy to Break the Step‐Reduction Delivery Dilemma
Source: Adv Sci (Weinh). 2022 Jun 26;9(24):2201819. doi: 10.1002/advs.202201819 (PMC9404413; doi:10.1002/advs.202201819)
Supplement: Supplementary file 1 — Supporting Information [file ADVS-9-2201819-s001.pdf]

ADVANCED SCIENCE
Open Access

Supporting Information

for *Adv. Sci.*, DOI 10.1002/advs.202201819

Targeting Bone Tumor and Subcellular Endoplasmic Reticulum via Near Infrared II Fluorescent Polymer for Photodynamic-Immunotherapy to Break the Step-Reduction Delivery Dilemma

Xianghong Zhang, Jia Wan, Fuhao Mo, Dongsheng Tang, Haihua Xiao, Zhihong Li, Jinpeng Jia* and Tang Liu**

Supporting Information

Targeting Bone Tumor and Subcellular Endoplasmic Reticulum via Near Infrared II Fluorescent Polymer for Photodynamic-Immunotherapy to Break the Step-Reduction Delivery Dilemma

*Xianghong Zhang*1,2, *Jia Wan*1,2, *Fuhao Mo*5, *Dongsheng Tang*2, *Haihua Xiao*2, *Zhihong Li**1,3, *Jinpeng Jia**4, *Tang Liu**1

1. Department of orthopedics, The Second Xiangya Hospital, Central South University, Changsha, Hunan, 410011, P. R. China.
2. Beijing National Laboratory for Molecular Sciences, State Key Laboratory of Polymer Physics and Chemistry, Institute of Chemistry, Chinese Academy of Sciences, Beijing, 100190, P. R. China.
3. Hunan Key Laboratory of Tumor Models and Individualized Medicine, The Second Xiangya Hospital, Central South University, Changsha, Hunan, 410011, P.R. China.
4. Senior Department of Orthopedics, the Fourth Medical Center of PLA General Hospital, Beijing, 100853, P. R. China.
5. State Key Laboratory of Advanced Design and Manufacture for Vehicle Body, Hunan University, Changsha, Hunan, 410082, P. R. China.

TABLE OF CONTENTS

Materials and Methods....1
Scheme S1....10
Scheme S2....11
Scheme S3....12
Figure S1....13
Figure S2....14
Figure S3....15
Figure S4....16
Figure S5....17
Figure S6....18
Figure S7....19
Figure S8....20
Figure S9....21
Figure S10....22
Figure S11....23
Figure S12....24
Figure S13....25
Figure S14....26
Figure S15....27
Figure S16....28
Figure S17....29
Figure S18....30
Figure S19....31
Figure S20....32
Figure S21....33
Figure S22....34
Figure S23....35
Figure S24....36
Figure S25....37
Table S1....38
References....39

1. Supplemental materials and methods

1.1. Materials and agents

3,3'-(((((5,6-dinitrobenzo[c][1,2,5]thiadiazole-4,7-diyl)bis(thiophene-5,2-diyl))bis((4-(1,2,2-triphenylvinyl)phenyl)azanediyl))bis(4,1-phenylene))bis(oxy))bis(propan-1-ol) ($C_{84}H_{64}N_6O_8S_3$, M_{AIE}) was synthesized as previously described.[1] Methoxypolyethylene glycol ($mPEG_{5000}$-OH), N-Tosylethylenediamine (T^{ER}), alendronic acid, hydroxylapatite, and 1,3-diphenylisobenzofurane (DPBF) were purchased from Aladdin (Shanghai, China). Other reagents were purchased from Energy Chemical *Co., Ltd.* (Shanghai, China). All chemicals were obtained from commercial sources and used without further purification unless otherwise noted.

Cell culture vessels were purchased from Corning (Corning, NY, USA). Dulbecco's modified Eagle's medium (DMEM) with 4.5 g glucose, RPMI-1640, trypsin-EDTA (0.25%), penicillin/streptomycin (P/S) and fetal bovine serum (FBS) were purchased from Gibco (Gran Island, NY, USA). 3-(4,5-dimethylthiazol-2-yl)-2,5-diphenyltetrazolium bromide (MTT) were purchased from Aladdin (Shanghai, China). 2-(4-amidinophenyl)-1H-indole-6-carboxamidine (DAPI), propidum iodide (PI), Alexa fluor 488, TUNEL Apoptosis Assay Kit was purchased from Solarbio Science & Technology *Co., Ltd.* (Beijing, China). Annexin V-FITC apoptosis detection kit was purchase from Elabscience. ER-Tracker Green and Fluo-3 AM were purchase from Beyotime (Shanghai, China). Antibodies used for flow cytometry analysis and immunostaining were listed in the supplementary Table S1.

1.2. General measurements

^{1}H NMR spectra was measured by a 400 MHz NMR spectrometer (Bruker, Germany) at room temperature. The morphology and size of nanoparticles were obtained by transmission electron microscope (TEM) carried out with a HT7700 (Hitachi, Japan). The absorption spectrum was measured using an ultraviolet-visible spectrometer (UV-vis, UV-2600, Shimadzu, Japan). Dynamic light scattering (DLS) was used to characterize the size of the self-assemble nanoparticles on a Malvern Zetasizer (Nano ZS, UK). Localization of nanoparticles and all the immunofluorescence slides were pictured through a confocal laser scanning microscope (CLSM) (LSM 800, ZEISS, Germany, and FLIM + confocal + AFM, ZEISS). The free radial detection and MTT assay was conducted using a Microplate reader (SpectraMax, USA). *In vivo* imaging was conducted by an *In Vivo* Imaging System (IVIS, Perkin Elmer, USA). Flow cytometry (FCM) was done with a CytoFLEX Flow Cytometry (Beckman Coulter, USA).

1.3. Cell culture

K7M2 cells were cultured in DMEM (glucose 4.5 g/L) supplemented with 10% fetal bovine serum and 1% P/S at 37 °C with 5% CO_2, MNNG/HOS and 143B cells were cultured in RPMI-1640 supplemented with 10% fetal bovine serum and 1% P/S at 37 °C with 5% CO_2. When the degree of cell fusion reached 80%-90%, the cells were digested with 0.25% trypsin, and then sub-cultured or inoculated in cell plates for subsequent experiments.

1.4. Methods

1.4.1. Synthesis of photodynamic and ROS sensitive polymer (P^{PDT})

The monomer of M_{AIE} was synthesized as previously described.[1] To a solution of above dihydroxy monomer (20.0 mg, 0.014 mmol) and reactive oxygen species (ROS) sensitive linker (2,2'-(propane-2,2-diylbis(sulfanediyl)) bis (ehan-1-ol) (M_1) (11.0 mg, 0.056 mmol) in anhydrous DMF (5 mL) was quickly added 1,2,4,5-cyclohexanetetracarboxylic dianhydride (M_2) (16.6 mg, 0.074 mmol). After magnetic stirring for another 8 h at room temperature, Methoxypolyethylene glycol ($mPEG_{5000}$-OH) (0.0074 mmol, 37 mg) was added to the reaction mixture. After magnetic stirring for another 12 h at 50 °C, the mixture was added into 15 mL of deionized water under sonication, followed by dialysis in a dialysis bag (MWCO: 8000-14000 Da). After 72 h, the solution was freeze-dried under reduced pressure to obtained 22 mg polymer (P^{PDT}).

1.4.2. Synthesis of photodynamic, ROS sensitive, and ER-targeting polymer ($P^{ER\text{-}PDT}$)

To a solution of P^{PDT} (22.0 mg) and EDC (5.0 mg) in DMF (5 mL) was added NHS (3.0 mg). After magnetic stirring for another 30 min at room temperature, T^{ER} (5.0 mg) was added to the reaction mixture. After magnetic stirring for another 12 h at room temperature, the mixture was added into 15 mL of deionized water under sonication, followed by dialysis in a dialysis bag (MWCO: 8000-14000 Da). After 72 h, the solution was freeze-dried under reduced pressure to obtained 22 mg polymer ($P^{ER\text{-}PDT}$).

1.4.3. Synthesis of photodynamic, ROS sensitive, and bone targeting polymer ($P^{BO\text{-}PDT}$)

To a solution of P^{PDT} (22.0 mg) and EDC (5.0 mg) in DMF (5 mL) was added NHS (3.0 mg). After magnetic stirring for another 30 min at room temperature, alendronic acid (5.0 mg) was added to the reaction mixture. After magnetic stirring for another 12 h at room temperature, the mixture was added into 15 mL of deionized water under sonication, followed by dialysis in a dialysis bag (MWCO: 8000-14000 Da). After 72 h, the solution was freeze-dried under reduced pressure to obtained 22 mg polymer ($P^{BO\text{-}PDT}$).

1.4.4. Formulation of NP^{PDT}, $NP^{ER\text{-}PDT}$ and $NP^{ER/BO\text{-}PDT}$

Briefly, a stirred solution of P^{PDT} (18.0 mg) in DMF (1 mL) was dropwise added to deionized water (9 mL). The above liquid was dialyzed in a dialysis bag (MWCO: 3500 Da) for 24 h. NP^{PDT} was obtained. $NP^{ER\text{-}PDT}$ was obtained in the same way as NP^{PDT}. A stirred solution of $P^{ER\text{-}PDT}$ (9.0 mg) and $P^{BO\text{-}PDT}$ (9.0 mg) in DMF (1 mL) was dropwise added to deionized water (9 mL). The above liquid was dialyzed in a dialysis bag (MWCO: 3500 Da) for 24 h, $NP^{ER/BO\text{-}PDT}$ was obtained.

1.4.5. Particle size and morphology, and storage stability

The size distribution of nanoparticles was detected by DLS at the concentration of photosensitive unit at 80 μg mL^{-1} in aqueous solution, and the morphology of nanoparticles was

observed using TEM. The concentrated aqueous solution of $NP^{ER/BO-PDT}$ was diluted with PBS. At the designated time, aliquot of the solution was taken and monitored by DLS.

1.4.6. Detection of ROS generation in aqueous solution

Photo-triggered singlet oxygen generation ($^1O_2$) of the $NP^{ER/BO-PDT}$ was determined by using the chemical trapping method.[2] The 100 μg mL^{-1} $NP^{ER/BO-PDT}$ were mixed with 1 μg mL^{-1} DPBF at a volume ratio of 1:40, followed by the analysis using a Microplate reader (SpectraMax, USA). The decomposition rate of DPBF by $NP^{ER/BO-PDT}$ was recorded after different irradiation time durations (808 nm, 1.0 W cm^{-2}), the absorbance changes of DPBF at 415 nm was used to quantify decomposition rate.

1.4.7. Nanoparticles uptake in the cells and ER colocalization by CLSM

A cover slide was placed in the bottom of each well of a 24-well plate. Cells (1×10^5) in 1 mL medium were added to each well and incubated at 37 °C for overnight. Then, the cells were incubated with $NP^{ER/BO-PDT}$@Cy5.5 for different durations. Next, the culture medium was removed and washed with PBS for three time followed by incubation with fresh media. Subsequently, the cells were incubated ER-Tracker Green base on the manufacturer's instruction. Then, the cells were washed with PBS for three times. The cover slide was placed on the microslide and the cell nuclei were stained with DAPI. Subsequently, images were collected by CLSM (DAPI, λ_{ex} = 405 nm, NPs, λ_{ex} = 633 nm, ER-Tracker Green, λ_{ex} = 488 nm). The Origin software were used to analysis and draw the pictures.

1.4.8. Detection of Cell Endocytosis of Nanoparticles

Cell endocytosis of nanoparticles were measured and quantified with the using of FCM instrument. Cells were seeded into 12-well plate at 2×10^5 cells/well and incubated at 37 °C for overnight. The $NP^{ER/BO-PDT}$@Cy5.5 were added to each well and the wells without any treatment were performed as negative control. Afterwards, the cells were harvested and quantified using FCM at different durations. Furthermore, the cells were treated $NP^{ER/BO-PDT}$ with the same conditions as FCM analysis for NIR II fluorescence (DAPI, λ_{ex} = 405 nm, NPs, λ_{ex} = 808 nm, Actin-Tracker Green-488, λ_{ex} = 488 nm).

In addition, cell endocytosis and endocytosis inhibition of nanoparticles were measured on 3D tumor spheroids by CLSM. Firstly, 1% agarose gel solution (50 μL) was added to each 96-well plate. Then, 1600 cells (200 μL complete medium) were added to each well. On the 7th day, the cell spheres were basically formed. Subsequently, the 3D spheroids were treated with $NP^{ER/BO-PDT}$@Cy5.5 at the same concentration. Some 3D spheroids for evaluation of cell endocytosis inhibition were treated with Genistein. Subsequently, images were collected by CLSM at different durations. Images were captured at intervals of 10 μm from top to bottom of the 3D tumor spheroids by a CLSM with Z-stack scanning.

1.4.9. *In vitro* cytotoxicity assays

The cytotoxicity of different treatments *in vitro* was measured by MTT assay, apoptosis analysis and live/dead cell staining of 2D or 3D tumor spheroids. The cells were divided into 7 groups for the cytotoxicity study: (1) PBS, (2) NP^{PDT}, (3) $NP^{ER\text{-}PDT}$, (4) $NP^{ER/BO\text{-}PDT}$, (5) NP^{PDT} + L, (6) $NP^{ER\text{-}PDT}$ + L, (7) $NP^{ER/BO\text{-}PDT}$ + L. For MTT assay, different OS cell lines (1×10^4 cells/well) were seeded into 96-well plate and further cultured with complete medium (10% FBS) at 37 ℃ for 24 h. Afterward, cells were incubated with different formulations and different concentrations (photosensitive unit concentrations ranging from 5 μg mL^{-1} to 40 μg mL^{-1}). After 12 h incubation, the cells in the laser groups were irradiated with NIR light of 808 nm at intensity of 1.0 W cm^{-2} for 3 min. Meanwhile, the dark cytotoxicity study was conducted on the nanoparticles incubated cells without light irradiation. After incubation for another 12 h, the viability was analyzed by MTT colorimetric assay. In brief, after total 24 h incubation, 10 μL MTT solution (5 mg mL^{-1} solution) was added into each well and the plates were further allowed to incubate with cells for another 4 h. Acidified SDS solution was then added (100 μL/well) and the plates were kept in the dark for an additional 12 h. Measurements of absorbance were subsequently made with a Bio-Rad plate reader (SpectraMax M3). Cellular apoptosis was assessed with an Annexin V-FITC apoptosis detection kit according to the manufacturer's instructions. In brief, cells were incubated with Annexin/PI reagent in the dark for 20 min at 25 ℃. Thereafter, the cells were immediately measured with FCM. For live/dead cell staining of 3D tumor spheroids, 1% agarose gel solution (50 μL) was added to each 96-well plate. Afterward, 1600 cells (200 μL complete medium) were added to each well. On the 7th day, the cell spheres were basically formed. The fresh complete medium containing different formulations was added after removing the culture medium. After 12 h incubation, the cells of the laser groups were irradiated with a NIR light of 808 nm at intensity of 1.0 W cm^{-2} for 3 min. After further incubation for another 12 h at 37 ℃, the 3D tumor spheroids were washed with PBS for three time and then were successively stained with PI base on the manufacturer's instruction of the live/dead cell staining kit, respectively. Last, the cells were imaged by CLSM with Z-stack scanning.

1.4.10. Intracellular ROS generation

Intracellular ROS level was investigated by using DCFH-DA as a fluorescent probe. In brief, a cover slide was placed in the bottom of each well of a 24-well plate. Cells (1×10^5) in 1 mL complete media were added to each well and incubated at 37 °C for 12 h. Afterward, the cells were treated with NP^{PDT}, $NP^{ER\text{-}PDT}$ and $NP^{ER/BO\text{-}PDT}$ at the same concentration of photosensitive unit for 8 h, respectively. The group of cells without any treatment were performed as negative control. Subsequently, the culture medium of the cells was replaced with a serum-free medium and then incubated with ROS indicator DCFH-DA (10 μM) for 20 mins. Then the cells of laser groups were irradiated with a NIR light at 808 nm after washing with PBS. Afterward, the cover slide of each well was placed on the microslide, and the cell nuclei were stained with DAPI. Subsequently, images were collected with CLSM. Furthermore, the intracellular ROS level was further detective and quantify by FCM. First, cells were seeded in 12-well plate at a density of 2×10^5 per well and incubated at 37 °C for 12 h. Afterward, the cells were treated with the same conditions as the above CLSM analysis. Finally, the cells were harvested to examine the intracellular DCFH-DA by FCM.

1.4.11. Measurements of cytosolic Ca^{2+}

For evaluation of the steady state cytosolic Ca^{2+}, cells after different formulations treatments (with or without laser irradiation) were suspended in solution containing 2.5 μM Fluo-3/AM based on Fluo-3/AM Assay Kit (Beyotime, China) according to the manufacturer's instruction. Afterward, the cells were incubated in the dark for 30 min at 37 ℃, washed by centrifugation and analyzed by FCM, and cytosolic Ca^{2+} level was expressed as the mean Fluo-3 fluorescence intensity. Furthermore, the steady state cytosolic Ca^{2+} was further detected by CLSM analysis. In brief, a cover slide was placed in the bottom of each well of a 24-well plate. Cells (1×10^5) in 1 mL complete media were added to each well and incubated at 37 °C for 12 h. Subsequently, the cells were treated with the same conditions as FCM analysis base on the manufacturer's instruction. Then, the cover slide was placed on the microslide, and the cell nuclei were stained with DAPI. Subsequently, images were collected with CLSM.

1.4.12. ER stress analysis

The ER stress was analyzed using western blot test to quantify five unfolded protein response (UPR): CCAAT-enhancer-binding protein homologous protein (CHOP), Phosphorylation of the inositol requiring enzyme-1α (p-IRE-1α), activating transcription factor 6 (ATF-6), spliced X-box Binding Protein-1 (XBP-1s), and Phosphorylation eukaryotic translation inhiation factor 2α (p-eIF-2α). The cells were treated with different formulations, and the cells of laser groups were expose to NIR light at 808 nm with an intensity of 1.0 W cm^{-2}. After a total 24 h incubation, the cells were harvested and lysed in RIPA (Radio-Immunoprecipitation Assay) Buffer (Sigma-Aldrich) containing Protease Inhibitor Cocktail (Sigma-Aldrich). The protein was quantified by the Coomassie brilliant blue protein quantitaion method. The primary antibodies used anti-p-DDIT3/CHOP (AF6684), anti-p-IRE-1α (AF5842), anti-ATF-6 (AF6243), anti-p-eIF-2α (AF6771) and anti-Vinculin (AF8313) were purchased from Beyotime Institute of Biotechnology. The primary antibodies used anti-XBP-1s (E9V3E) was purchased from Cell Signaling Technology. Biotin-labeled goat anti-rabbit IgG (A0277) secondary antibodies were obtained from Beyotime Institute of Biotechnology. The detailed process can refer to the steps of western blot test.

1.4.13. Immunogenic cell death *in vitro*

To determine different formulations-induced immunogenic cell death (ICD) of the tumor cells, secretion of adenosine triphosphate (ATP), calreticulin (CRT) exposure, and extracellular release of high mobility group box 1 (HMGB1) were examined *in vitro*. Extracellular secretion of ATP was evaluated with a commercially available ATP assay kit. Briefly, cells were seeded in 12-well plate at a density of 2×10^5 cells/well. After incubated for 12 h, the cells were treated with PBS, NP^{PDT}, $NP^{ER\text{-}PDT}$, $NP^{ER/BO\text{-}PDT}$, NP^{PDT} + L, $NP^{ER\text{-}PDT}$ + L, and $NP^{ER/BO\text{-}PDT}$ + L at an equal photosensitive unit concentration for 24 h. Afterward, the culture medium was collected and the concentration of ATP was evaluated with an ATP assay kit according to manufacturer's instructions.

CRT exposure was evaluated by FCM and CLSM. For FCM analysis, cells were seeded in the 12-well plate at a density of 2×10^5 cells/well. After 12 h pre-incubation, the cells were treated

with PBS, NP^{PDT}, $NP^{ER\text{-}PDT}$, $NP^{ER/BO\text{-}PDT}$, NP^{PDT} + L, $NP^{ER\text{-}PDT}$ + L, and $NP^{ER/BO\text{-}PDT}$ + L at an equal photosensitive unit concentration for 6 h. Then the cells were washed with PBS and further incubated with CRT antibody. After that, the cells were incubated with secondary antibody, washed with PBS, collected and suspended in PBS. Finally, the surface fluorescence was assayed with FCM. For CLSM analysis, a cover slide was placed in the bottom of each well of a 24-well plate. Cells (1×10^5) in 1 mL complete media were added to each well and incubated at 37 °C for 12 h. Then the cells were treated with different formulations for 6 h like the FCM analysis. Next, the cells were fixed with 4% paraformaldehyde for 20 min, followed by incubation with 1% fetal bovine serum in PBS for 30 min. Then the cells were incubated with primary CRT antibody for night at 4 ℃, and then incubated with the 488-conjugated secondary antibody after three washes with PBS. Finally, the cells were stained with DAPI, and observed under CLSM using 405 nm and 488 nm lasers for visualizing nuclei and CRT exposure on the cell membrane, respectively. For evaluating intracellular HMGB1, a cover slide was placed in the bottom of each well of 24-well plate. Cells (1×10^5) in 1 mL complete media were added to each well. After 12 h pre-incubation, the cells were treated with different formulations like the CRT test for 24 h. After that, the cells were washed with PBS for three times. Then, the cells were fixed with 4% paraformaldehyde for 20 min and permeabilized with 0.1% Triton X-100 for 10 min, followed by incubation with 1% fetal bovine serum in PBS for 30 min. Next, the cells were incubated with primary HMGB1 antibody for night at 4 ℃, and then incubated with the 555-conjugated secondary antibody after three washes with PBS. Finally, the cells were stained with DAPI and examined by CLSM.

1.4.14. Dendritic cells activation *in vitro*

To evaluate dendritic cells (DCs) activation *in vitro*, bone marrow-derived dendritic cells (BMDCs) were obtained from the bone marrow of mice. K7M2 cells were first treated with PBS, NP^{PDT}, $NP^{ER\text{-}PDT}$, $NP^{ER/BO\text{-}PDT}$, NP^{PDT} + L, $NP^{ER\text{-}PDT}$ + L, and $NP^{ER/BO\text{-}PDT}$ + L at an equal photosensitive unit concentration for 24 h. Afterwards, BMDCs were cultured with 1.5 mL fresh medium and 1.5 mL tumor cells cultured medium obtained from different pretreated K7M2 cells groups. After that, the BMDCs were collected and stained with anti-CD11c, anti-CD80 and anti-CD86 antibodies. Finally, the activation of DCs was examined by using FCM measurement.

1.4.15. Western blotting

Cell lysate (RIPA: protease inhibitor: phosphorylated protease inhibitor = 100:2:1) was added to the cell dish to extract the all proteins in the cells. The protein was quantified by the Coomassie brilliant blue protein quantitaion method. An appropriate separation gel was selected according to the molecular weight. Twenty-five micrograms of protein sample was added per lane, and the samples were separated by SDS-PAGE gel electrophoresis, and then transferred to the polyvinylidene difluoride (PVDF) membrane. After blocking for one hour with 5% milk or 5% BSA, membranes were incubated with the primary antibody overnight at 4 °C, followed by incubation with the corresponding secondary antibody for one hour at room temperature. Membranes were visualized with a Tanon 5200 Multi Imaging System after incubation with an anti-mouse or rabbit secondary antibody (Beyotime Biotechnology).

1.4.16. Animal welfare and protocols

Healthy KM mice, BALB/c mice, and BALB/c nude mice were purchased from SPF Biotechnology (Beijing, China) and raised in SPF animal rooms. All animal experiments reported herein were performed under guidelines evaluated and approved by Peking University Institutional Animal Care and Use Committee (LA2021316).

1.4.17. *In vivo* biosafety evaluation

Healthy KM mice were randomly grouped (n = 3 mice per group). Each mouse was injected with a single dose of PBS, NP^{PDT}, $NP^{ER\text{-}PDT}$ and $NP^{ER/BO\text{-}PDT}$ (dose = photosensitive unit at 10 mg kg^{-1} body weight) through tail vain, respectively. The mice were monitored and weighed on alternate days. Then the mice were sacrificed at 14 days after administration, and the blood samples of the mice were collected for hematological and serum biochemical analyses. One blood sample of each group was used for blood routine examination, and another blood sample of each group was centrifuged at 3000 rpm for 10 min to collect plasma, which was further used for analysis of urea nitrogen (BUN), creatinine (CREA), alkaline phosphatase (ALP), total bilirubin (TBIL), aspartate aminotransferase (AST), and alanine transaminase (ALT). Meanwhile, the main organs were also dissected from the mice for further analysis after being sacrificed. All the tissues were paraffin embedded and tissue sections were prepared. Hematoxylin and eosin (H&E) staining was performed on the tissue sessions to observe pathological features, and images were captured using a fluorescence microscope (IX83, Olympus).

1.4.18. Establishment of K7M2 OS orthotopic model

K7M2 cells (3×10^6) were dispersed in PBS buffer and implanted into right tibia of BALB/c mice to establish an orthotopic tumor-bearing model. One week after the tumor was implanted, the tumor was grown to about 100 mm^3.

1.4.19. *In vivo* biodistribution imaging.

The $NP^{ER\text{-}PDT}$ and $NP^{ER/BO\text{-}PDT}$ (photosensitive unit 10 mg kg^{-1} body weight) was intravenously injected into the tumor-bearing mice. Afterward, the mice were anesthetized for *in vivo* biodistribution imaging at different interval time after injection. Biodistribution images were collected by IVIS (Spectrum CT, PerkinElmer, E_x/E_m=745 nm/840 nm) at various time points. The mice were sacrificed after 48 h of nanoparticles injection. *Ex vivo* imaging of organs including heart, liver, spleen, lung, kidney, intestine, and tumor were collected and quantitative analyses using IVIS Spectrum imaging system.

1.4.20. Histological and immunohistochemical analyses

After complete treatment, some mice were humanely sacrificed, followed by the excision of major organs and tumors for histological observation by standard H&E staining and immunofluorescence (IF) staining. For H&E staining, the excised tumor and organs were fixed in 4% paraformaldehyde solution, embedded in paraffin, sectioned and stained with

hematoxylin and eosin. The sections were then observed under a fluorescence microscope (IX83, Olympus). TUNEL staining was also used to evaluate apoptosis of tumor tissues, the procedures were consistent with the manufacturer's protocol, and finally examined by CLSM.

1.4.21. Immunogenic cell death *in vivo*

The BALB/c mice were orthotopically embedded K7M2 cells into right tibia. Once the tumor volume reached approximately 100 mm^3, the mice were assigned at random and received the same treatment as previously described. Mice were sacrificed after complete treatment and the blood samples of the mice were collected. The blood sample of each group was centrifuged at 3000 rpm for 10 min to collect plasma, which was further used for analysis of TNF-α, IFN-γ, and IL-10. In brief, the plasmas from each treatment were collected and then measured using assay kit according to manufacturer's instructions.

To detect the CRT exposure, the tumor tissue from each group were embedded in OCT and cross sectioned at 10 μm. These sections were incubated with the primary antibodies of anti-CRT overnight at 4 ℃ and the secondary Alexa Fluor 555-labeled goat anti-rabbit IgG (H + L) according to the manufacturer's protocols. By contrast, the sections were counterstained with DAPI as a control for CLSM visualization. For evaluating intracellular HMGB1 and the infiltration of $CD8^+$ T cells, the procedures were performed according to the manufacturer's protocols.

1.4.22. Flow cytometry analysis of the animal tissue

The BALB/c mice were orthotopically embedded K7M2 cells into tibia. Then the mice were assigned at random and received the different treatments as previously described, and the mice were sacrificed after completing treatment. The obtained fresh tumors, tumor-draining lymph nodes and spleens were used to prepare single-cell suspensions. The single-cell suspensions were further incubated with various antibodies against the immune cells. FCM analysis was carried out on the FCM machine, and the results were analyzed with the FlowJo_V10 software.

To detect the impacts on DCs maturation in tumor, the tumor tissues from each treatment were collected and homogenized into single cell suspension. The cell suspensions were respectively stained with primary antibodies of anti-CD11c-PE, anti-CD80-FITC and anti-CD86-APC for FCM analysis. The matured DCs were denoted as $CD11c^+CD80^+CD86^+$ cells. To detect the impact on DCs maturation in tumor-draining lymph nodes, the lymph nodes from each treatments were collected and homogenized to prepare single cell suspension, and the procedures were performed as above in tumor tissues. To detect the infiltration of antitumor T cells, the tumor tissues and spleens were cut into small pieces and homogenized into single cell suspension. To measure the tumor infiltration of $CD8^+$ T cells, the isolated cell suspensions were incubated with anti-PC5.5-CD3 or anti-PE-CD3, anti-CD8-FITC and anti-CD4-APC for FCM analysis. The $CD8^+$ T cells were marked as $CD3^+CD4^-CD8^+$ T cells, which was presented as the percentage of $CD4^-CD8^+$ cells in $CD3^+$ T cells. To detect the ability on eliminating M2 type TAMs and increasing M1 macrophages, the tumor tissues were cut into small pieces and homogenized into single cell suspension. Then the isolated cell suspensions were incubated with anti-F4/80-PE, anti-CD80-FITC and anti-CD206-APC according to the manufacturer's

protocols. The cell population of M2 phenotype TAMs (characterized as $CD80^{-}$ $CD206^{+}$ cells) and M1 phenotype macrophages (characterized as $CD80^{+}CD206^{-}$ cells) were determined using FCM analysis. Then, the M1/M2 ratio was calculated to assess their ability to restore the antitumor activities. Finally, Tregs ($CD4^{+}Foxp3^{+}$) in tumor tissues were also evaluated.

1.4.23. Establishment of PDX model of OS

The patient-derived OS xenograft anima model (PDX^{os}) was established in BALB/c nude mice. OS tissues were obtained from the Second Xiangya hospital of Central South University, with approval from the hospital's ethics committee and in compliance with all relevant ethical regulations (2020591). Patient-derived tumor of OS was freshly collected, cut into small fragment and transplanted subcutaneously into BALB/c nude mice.

1.4.24. Statistical analysis

Data were presented as mean ± standard deviation (SD). The data was analysis on GraphpadPrism 8. Student's t-test, one-way analysis of variance (ANOVA), and Kaplan-Meier method were performed. Differences were considered statistically significant at a level of $*p < 0.05$; $**p < 0.01$; $***p < 0.001$.

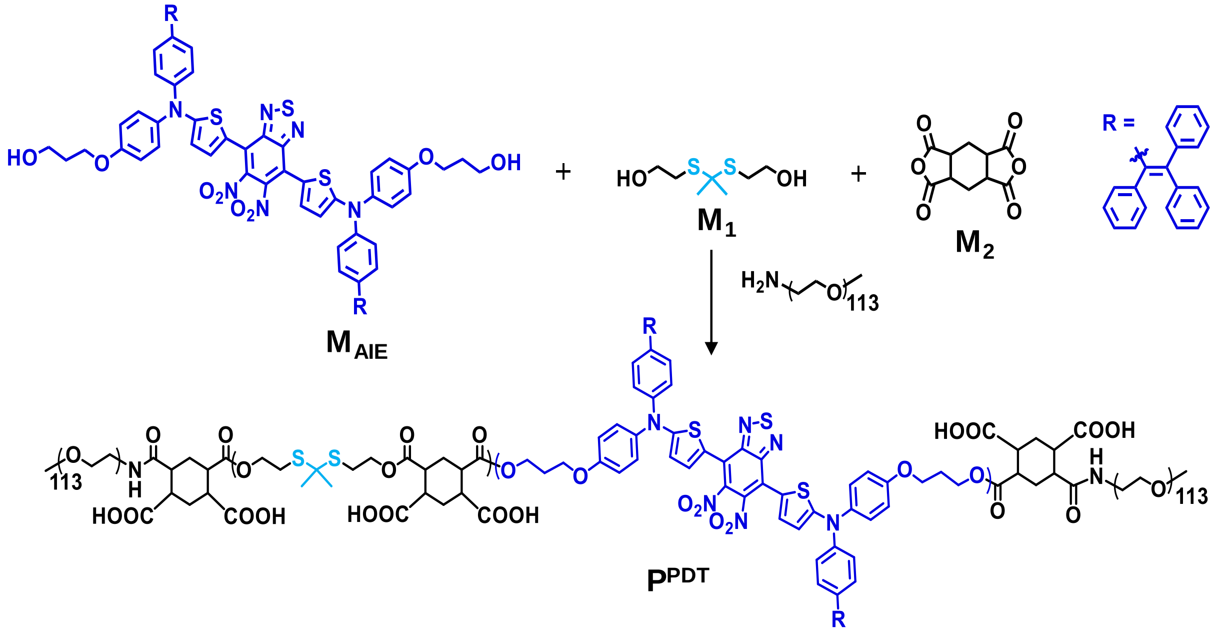

Scheme S1. Synthesis route of P^{PDT}. The monomer of M$_{AIE}$ was synthesized as previously described. [1]

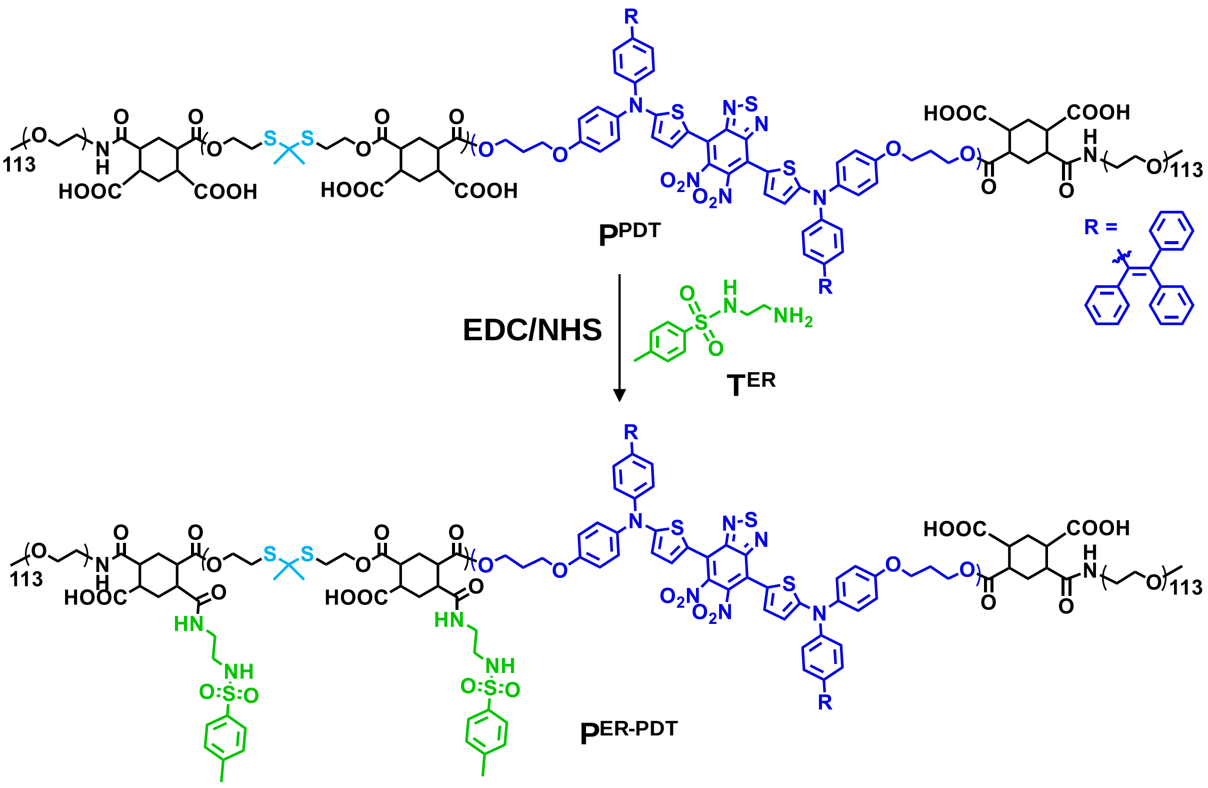

Scheme S2. Synthesis route of Endoplasmic reticulum (ER)-targeting polymer $P^{ER\text{-}PDT}$.

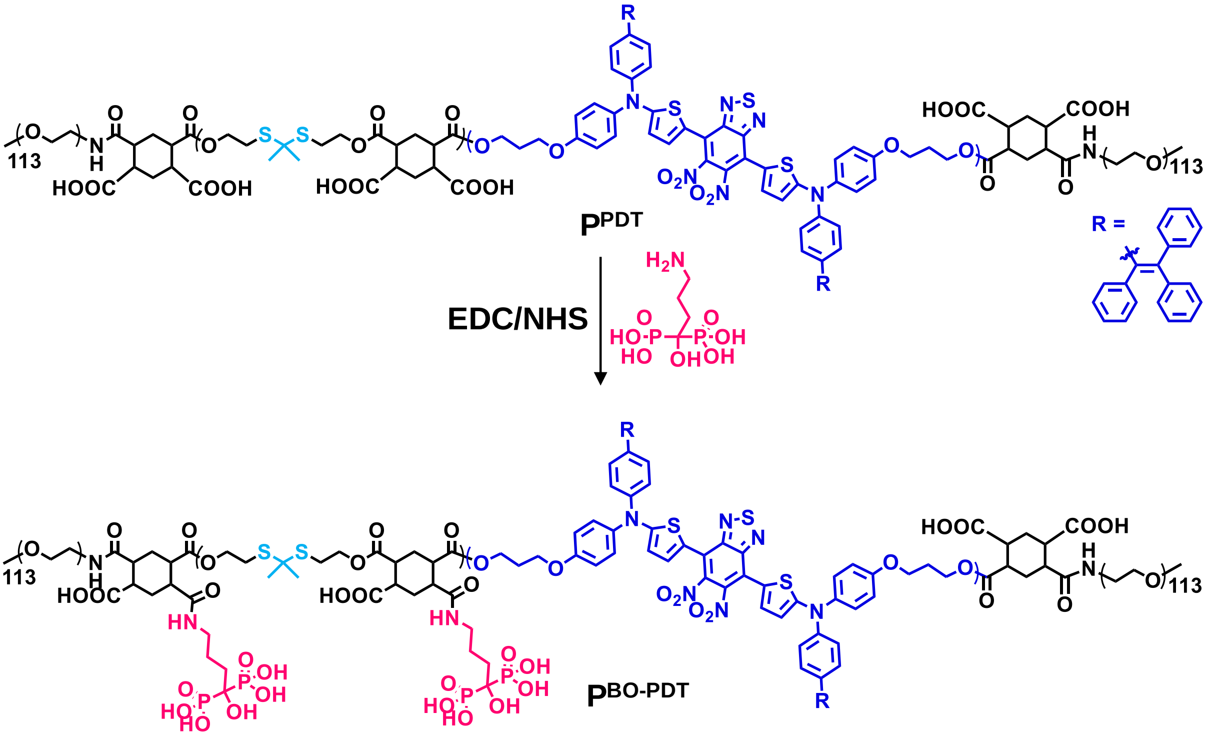

Scheme S3. Synthesis route of bone-targeting polymer P$^{BO\text{-}PDT}$.

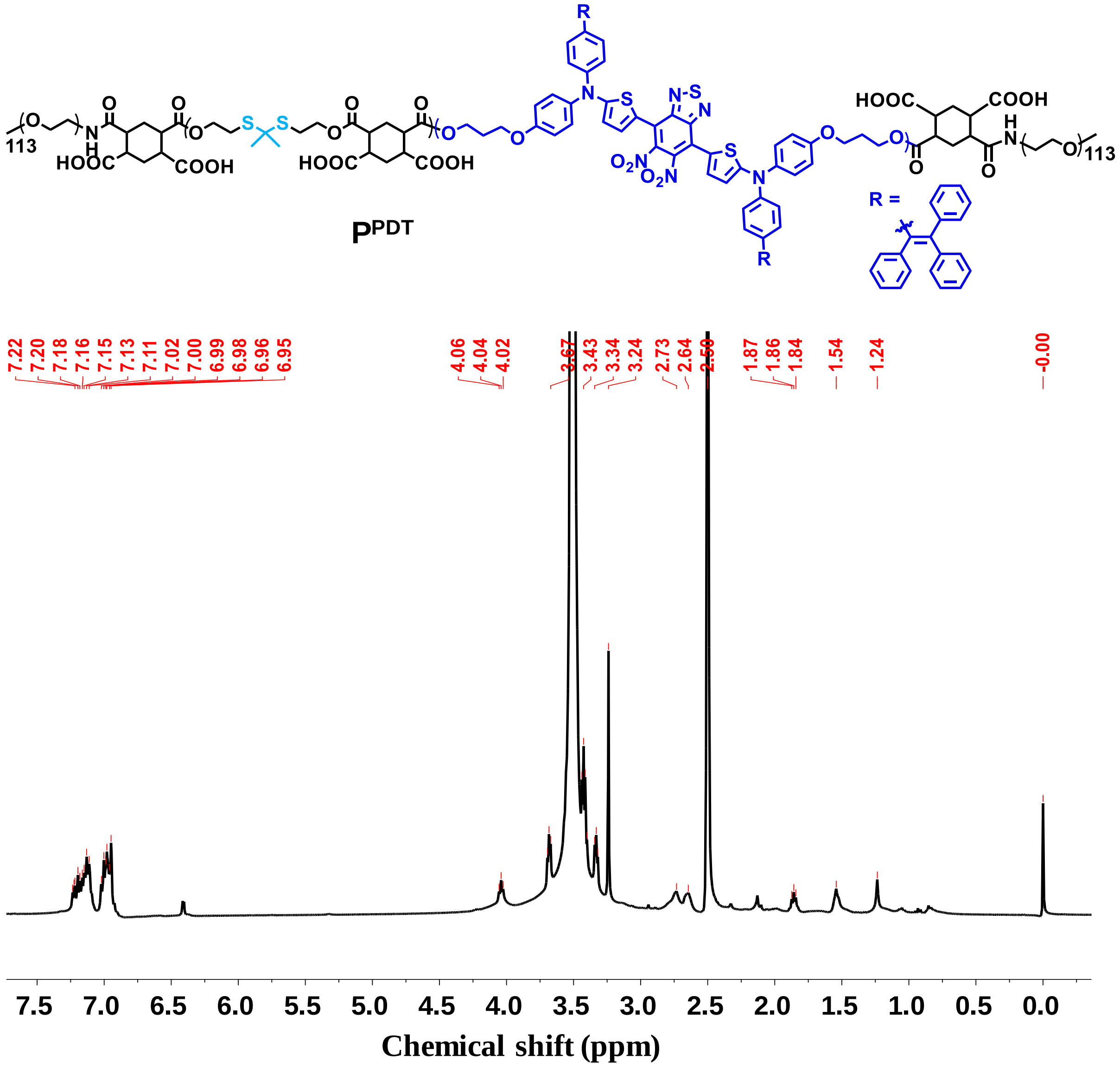

Figure S1. Characterization of P^{PDT} by ^1H NMR.

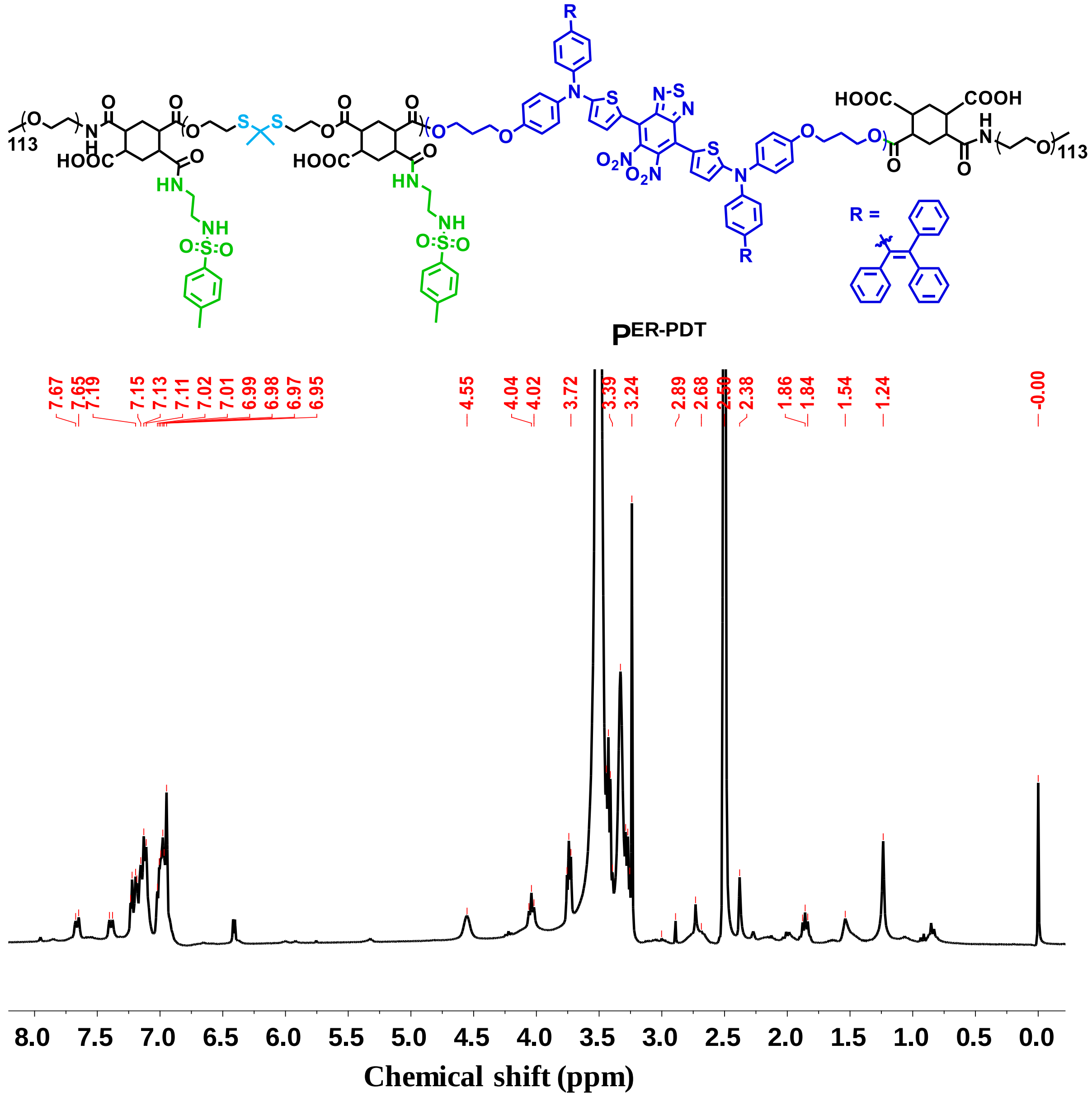

Figure S2. Characterization of $P^{ER\text{-}PDT}$ by ^{1}H NMR.

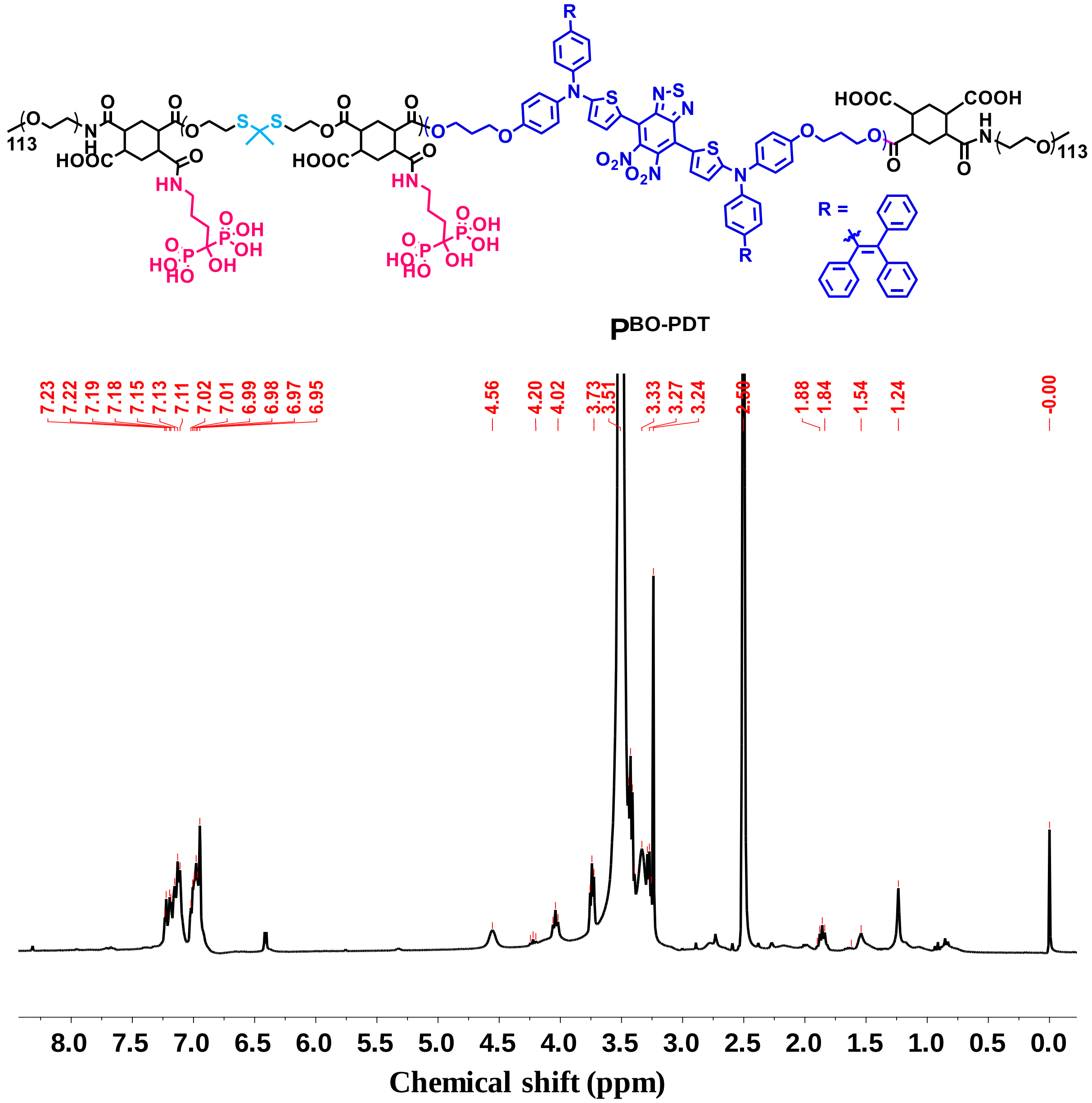

Figure S3. Characterization of $P^{BO\text{-}PDT}$ by ^{1}H NMR.

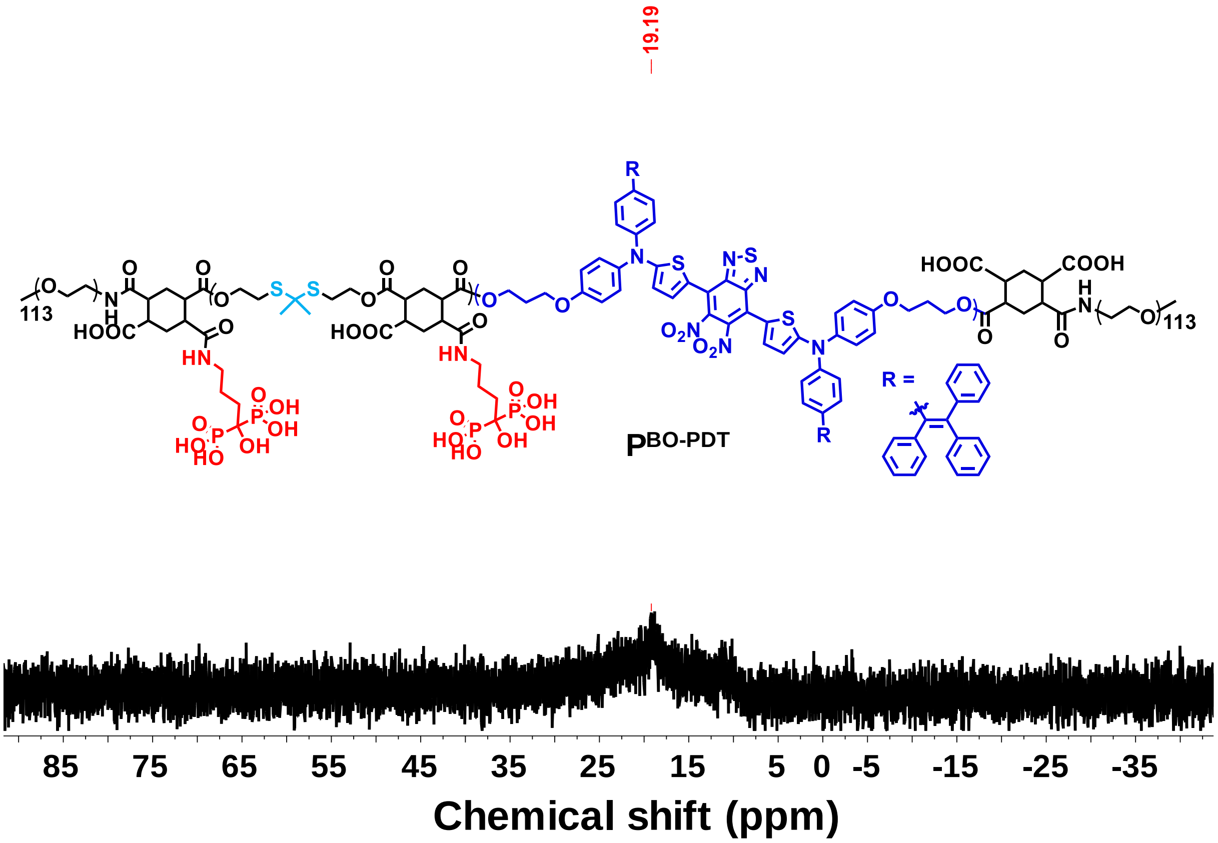

Figure S4. Characterization of P$^{BO\text{-}PDT}$ by ^{31}P NMR.

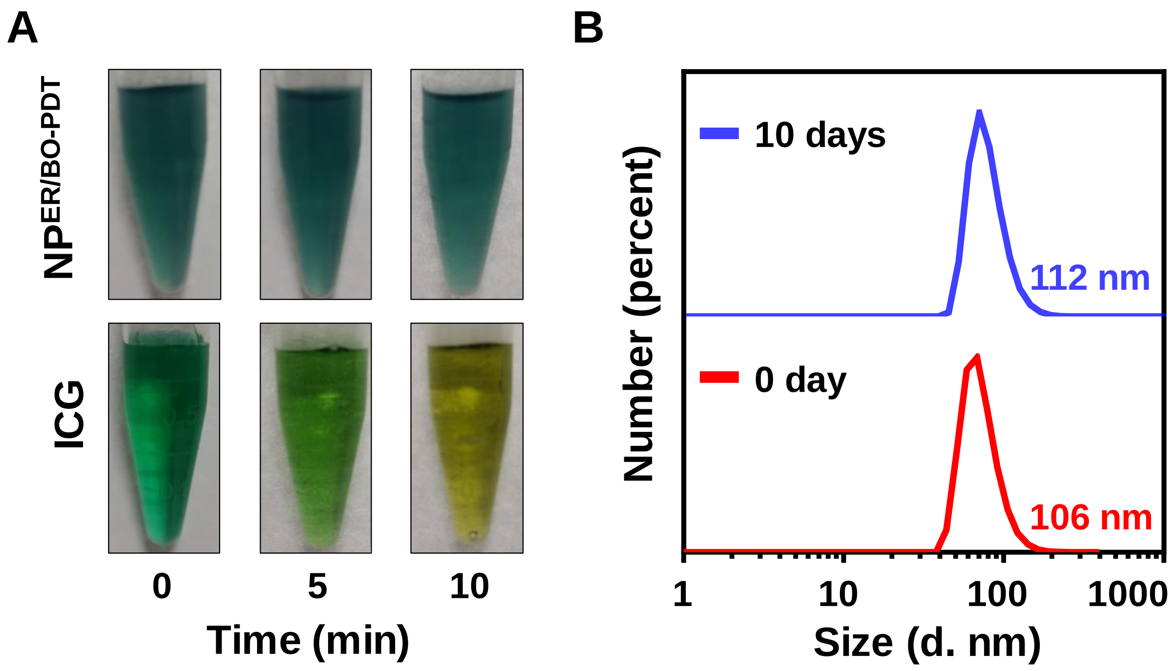

Figure S5. Characterization of $NP^{ER/BO\text{-}PDT}$. A) The changes of color of $NP^{ER/BO\text{-}PDT}$ and ICG under NIR light irradiation (808 nm, 1.0 W cm^{-2}) at different timepoints. B) Diameters of $NP^{ER/BO\text{-}PDT}$ after 10 days of dissolution in PBS by DLS.

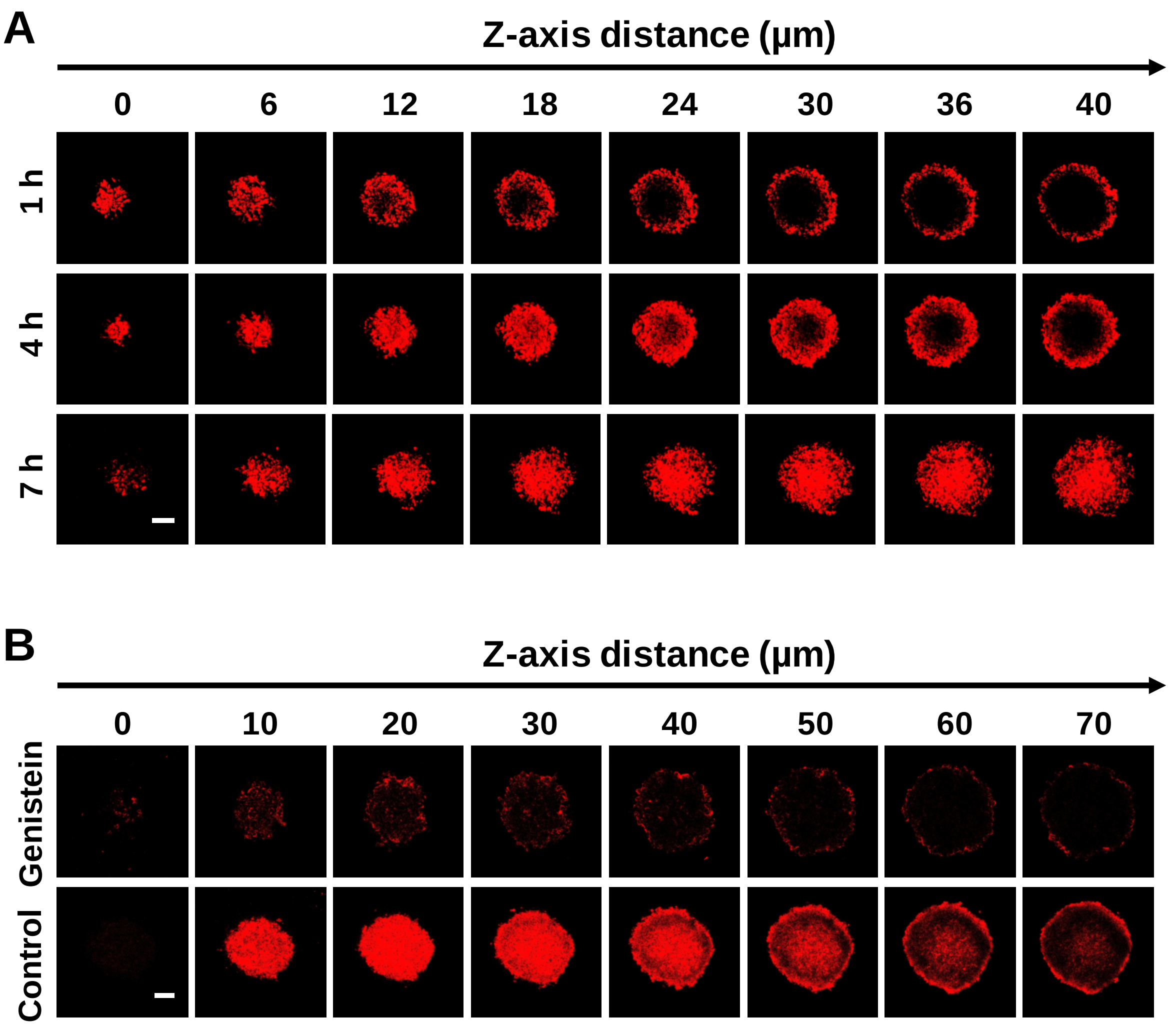

Figure S6. Representative CLSM images of Cy5.5 labeled NP$^{ER/BO-PDT}$ by 3D spheroids of K7M2. A) CLSM images of the intracellular uptake at different incubation times, and B) CLSM images of the intracellular uptake after treating with Genistein. Scale bar: 100 μm.

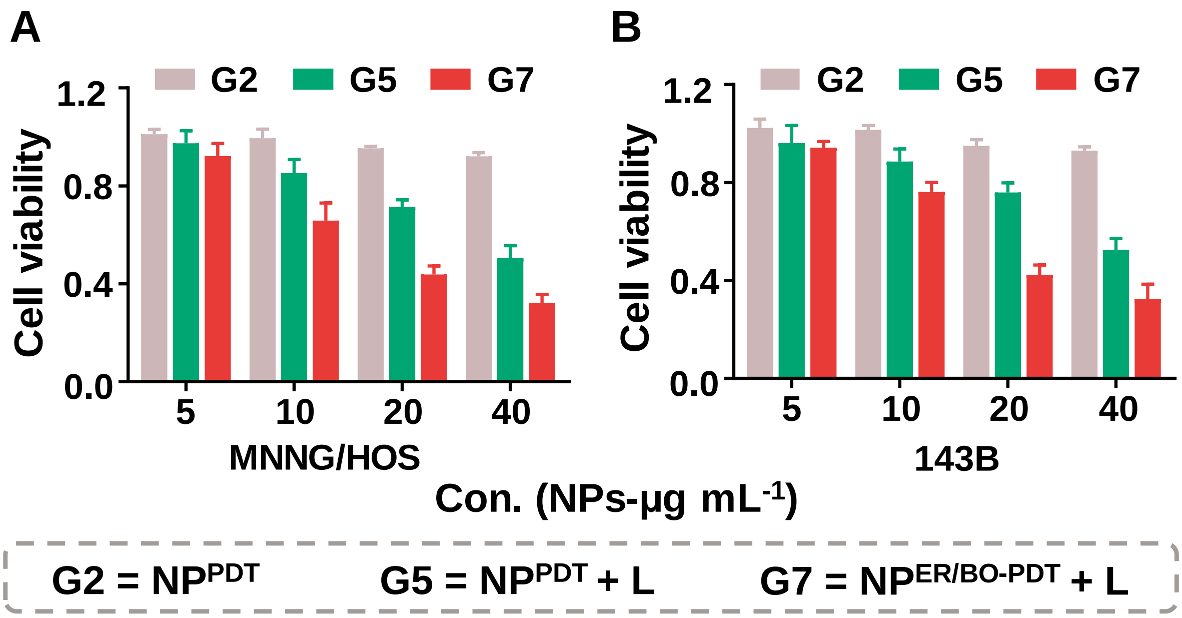

Figure S7. Under NIR light irradiation (808 nm, 1.0 W cm^{-2}, 3 min), $NP^{ER/BO-PDT}$ could enhance the cytotoxicity effect. A) Cell Viability of MNNG/HOS cells and B) 143B cells following various treatments with or without NIR light irradiation.

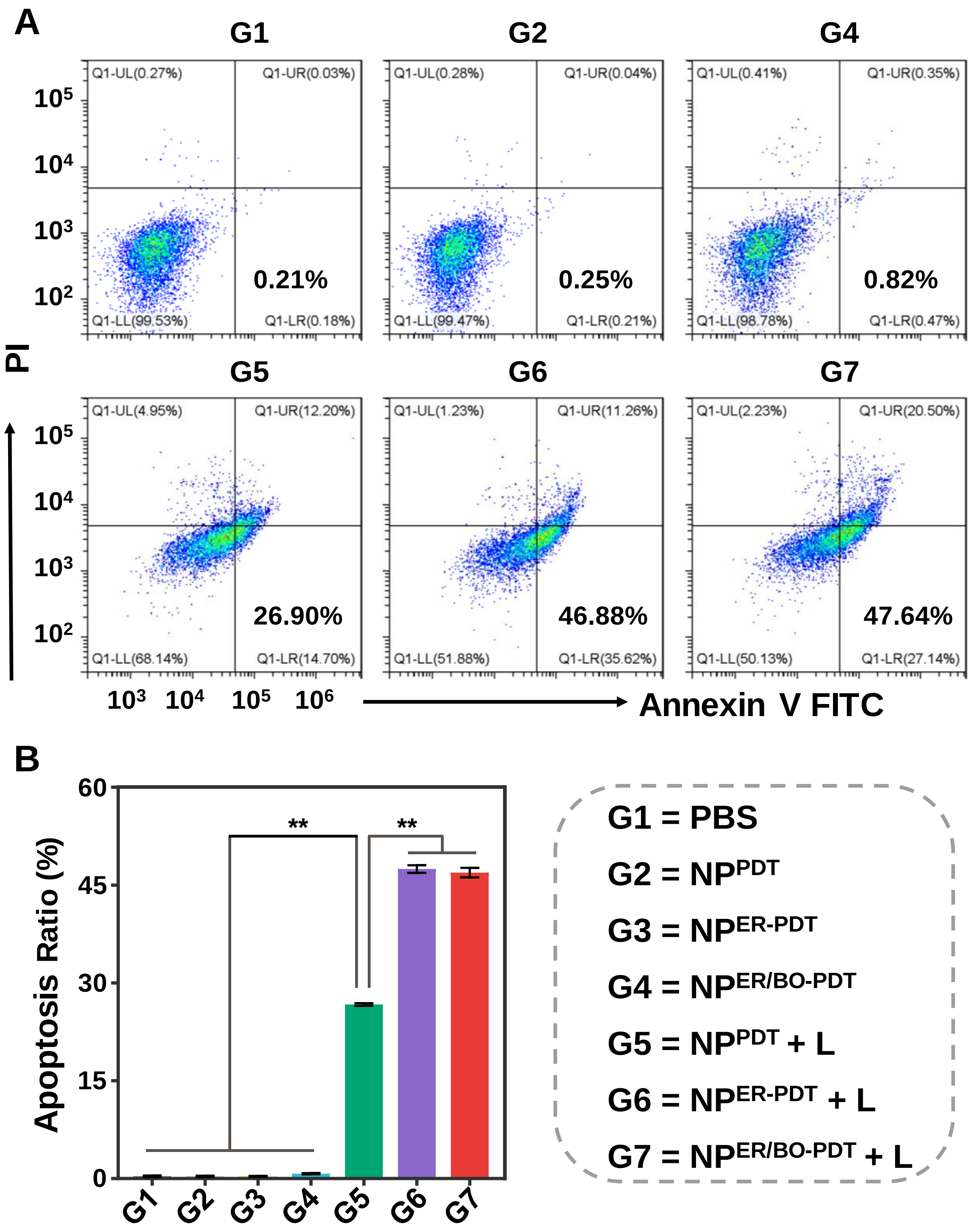

Figure S8. Cell apoptosis of K7M2 cells determined by FCM. A) FCM analysis and B) corresponding quantification of the apoptosis ratio after various treatments. Data are shown as mean ± SD. B) Student's *t*-test. $^{**}p < 0.01$.

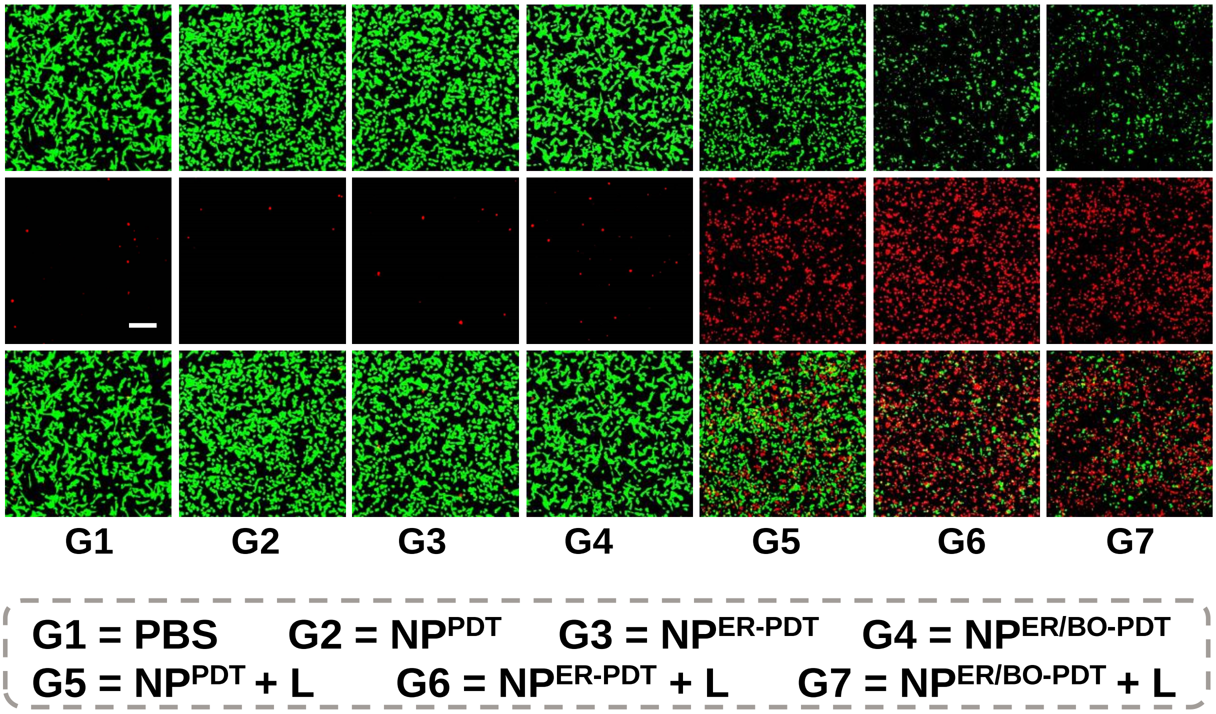

Figure S9. The CLSM images of MNNG/HOS stained with Calcein-AM (green, viable) and PI (red, dead), and the cells were treated at the same concentration of photosensitive unit. Scale bar: 200 μm.

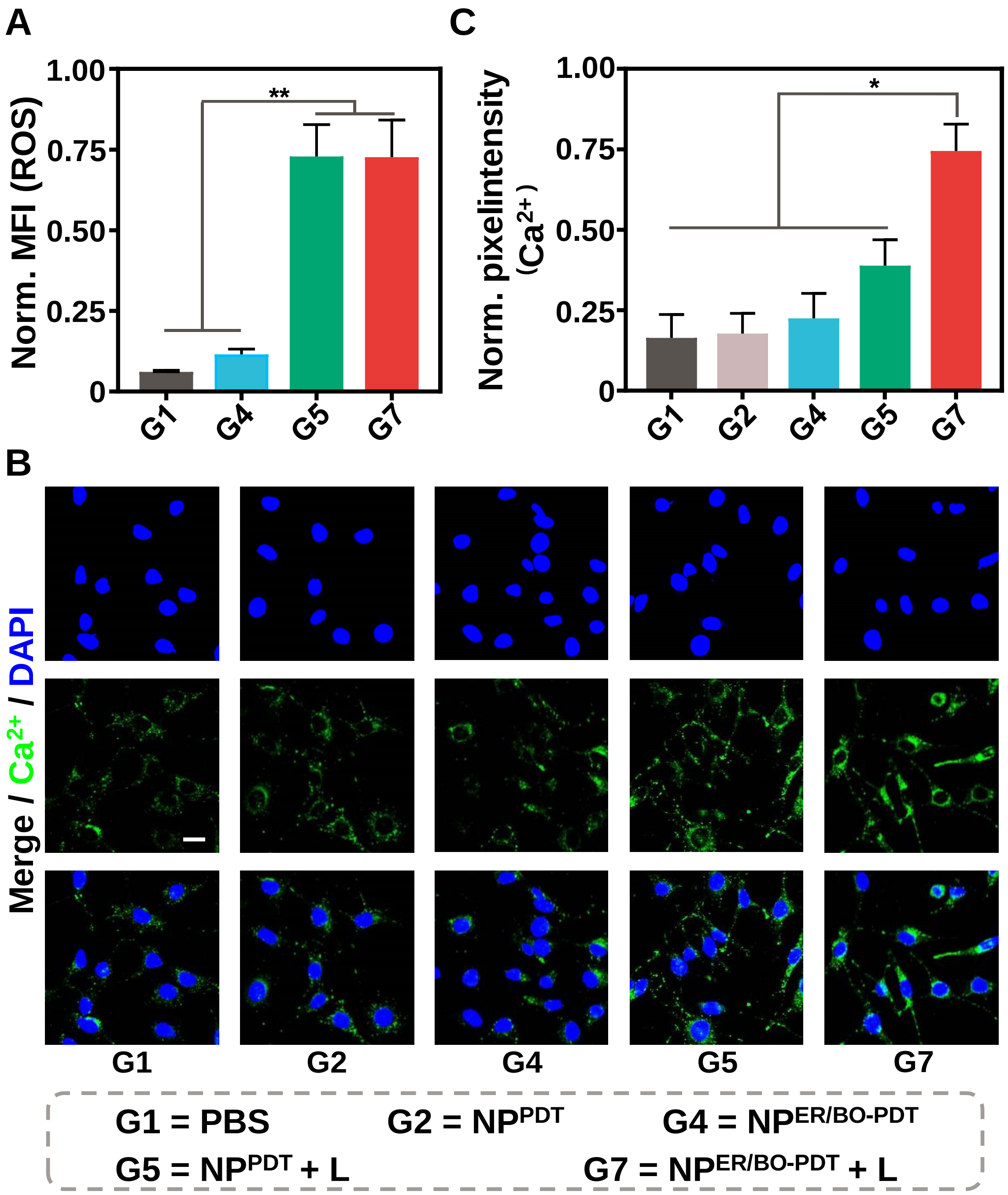

Figure S10. NP$^{ER/BO-PDT}$ induced the imbalance of Ca^{2+} in the ER lumen by the generated ROS. A) The mean florescence intensity (MFI) of intracellular ROS by FCM. B) CLSM images showed the distribution of intracellular Ca^{2+}. Scale bar: 20 μm. C) The relative MFI of intracellular Ca^{2+} by CLSM with Image J software. Data are shown as mean ± SD. A, C) Student's *t*-test. $^{*}p < 0.05$, $^{**}p < 0.01$.

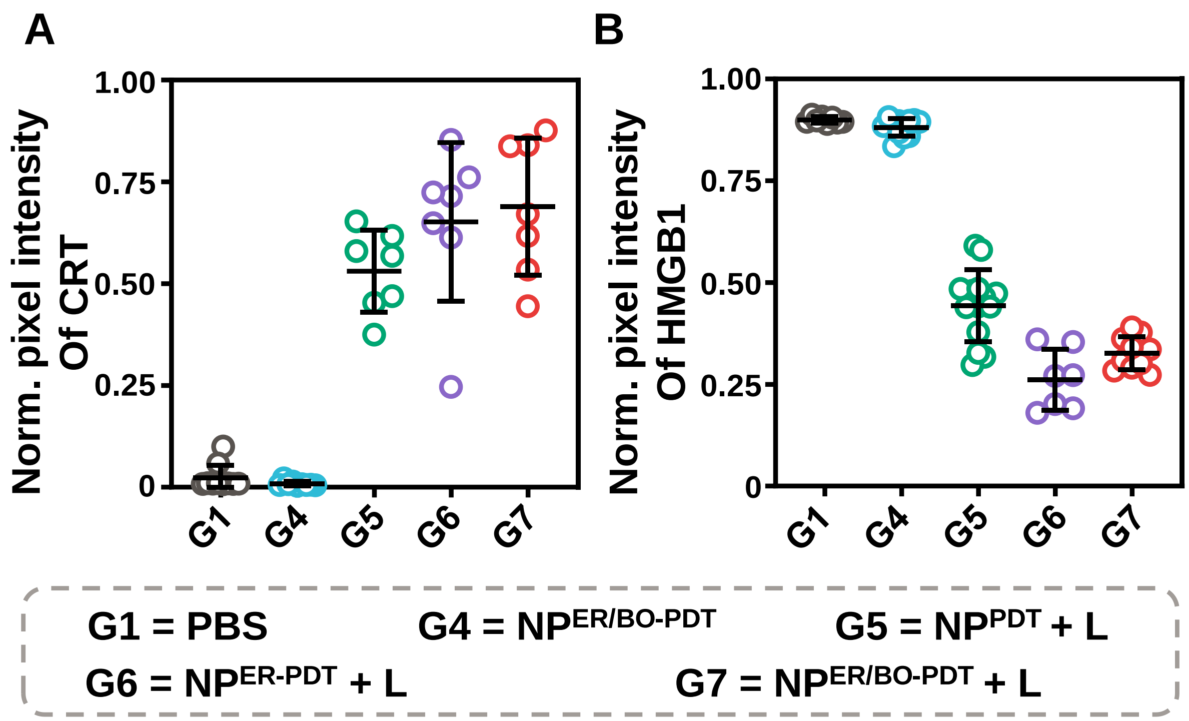

Figure S11. Quantification of the released DAMPs induced by NP$^{ER/BO-PDT}$ under NIR light irradiation by Image J software. A) Quantification of CRT exposure in Figure 3D by pixel intensity, and B) Quantification of HMGB1 release in Figure 3F by pixel intensity.

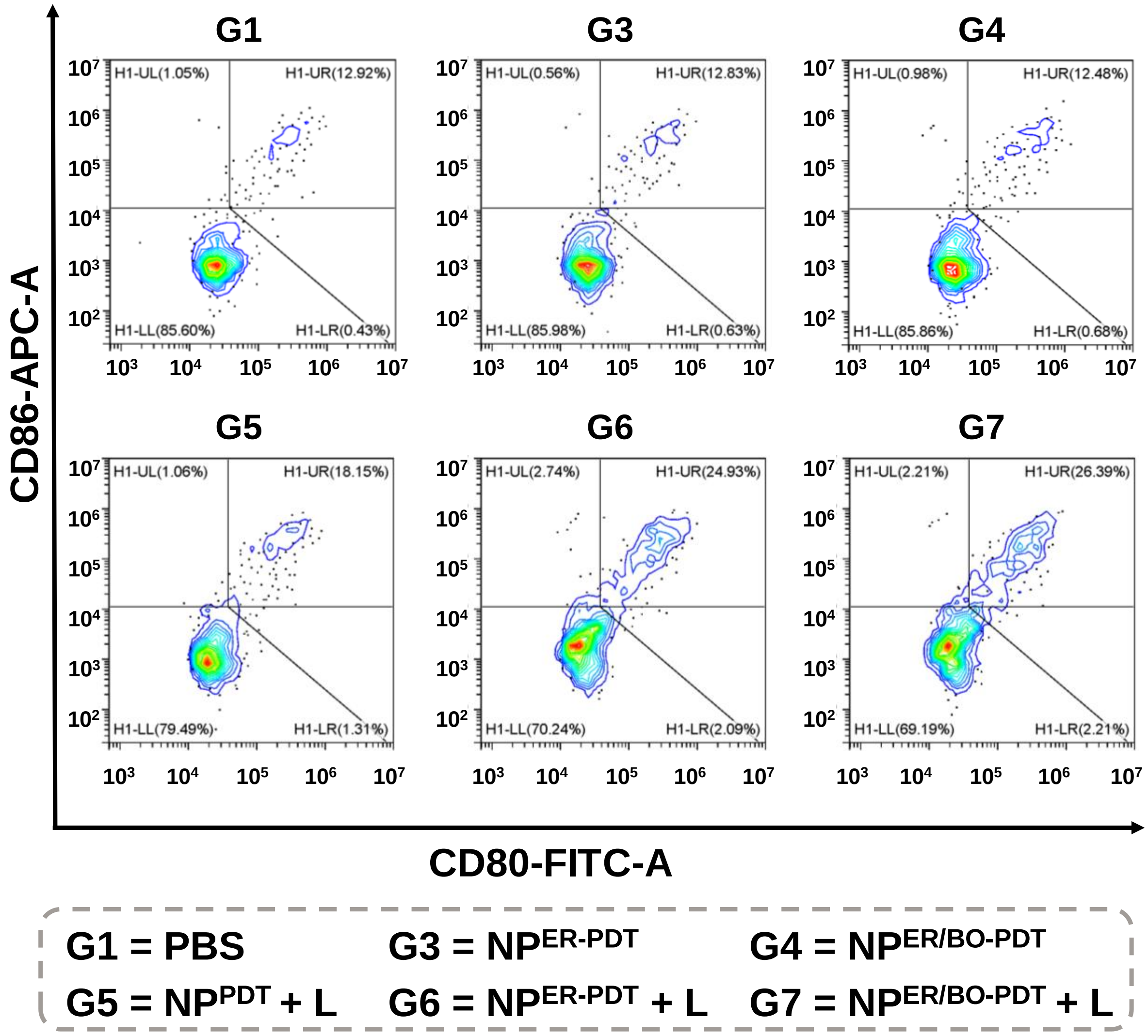

Figure S12. Representative flow cytometry plots indicate the proportions of $CD80^{+}CD86^{+}$ DCs after co-cultured with K7M2 cells with various pretreatments.

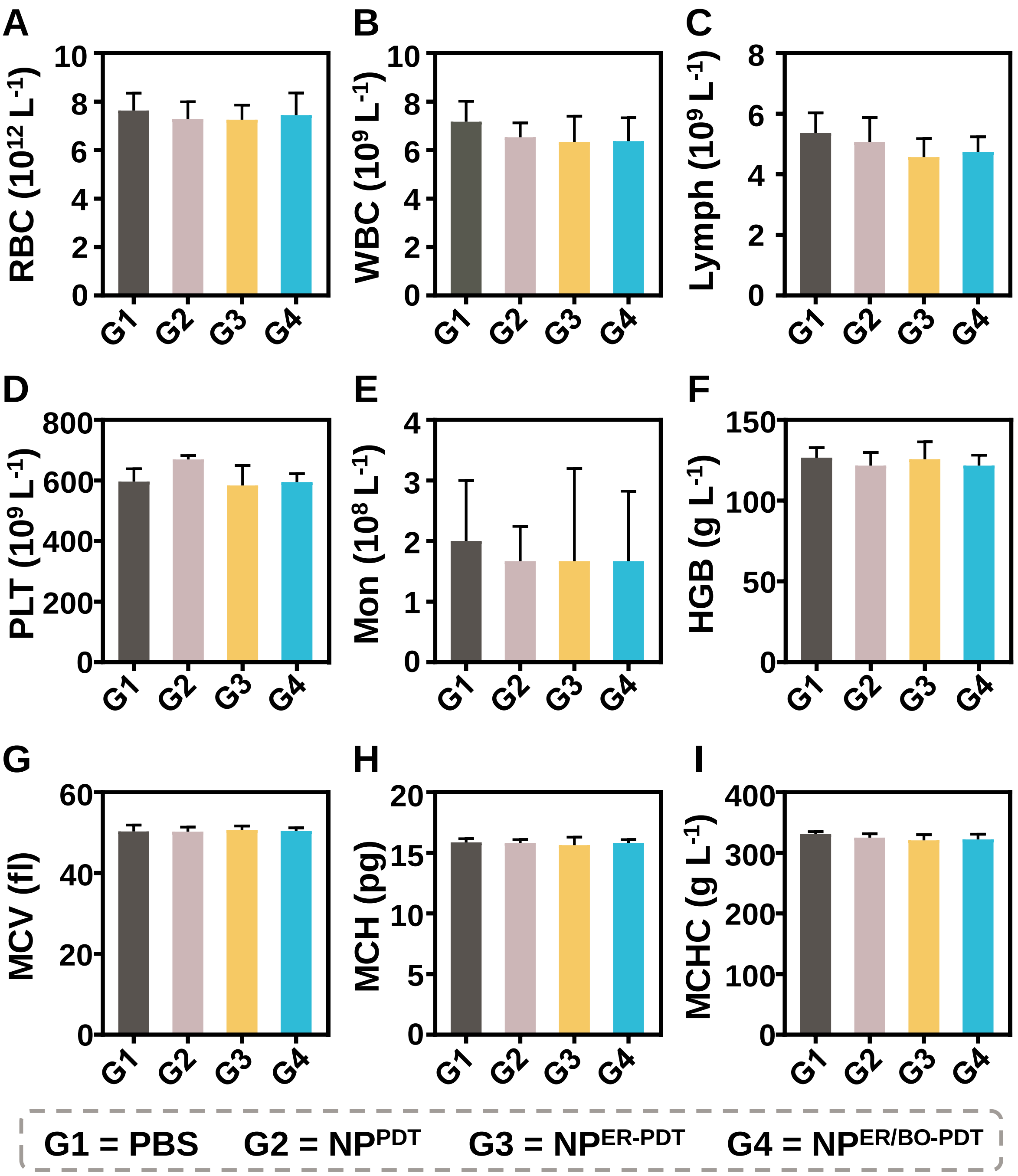

Figure S13. Biosafety evaluation of NP$^{ER/BO-PDT}$ *in vivo*. A-H) Biochemical analysis of whole blood: A) red blood cells (RBC); B) white blood cells (WBC); C) lymphocyte; D) blood platelet (PLT); E) Monocyte (Mon); F) hemoglobin (HGB); G) mean corpuscular volume (MCV); H) mean corpuscular hemoglobin (MCH); I) mean corpuscular hemoglobin concentration (MCHC).

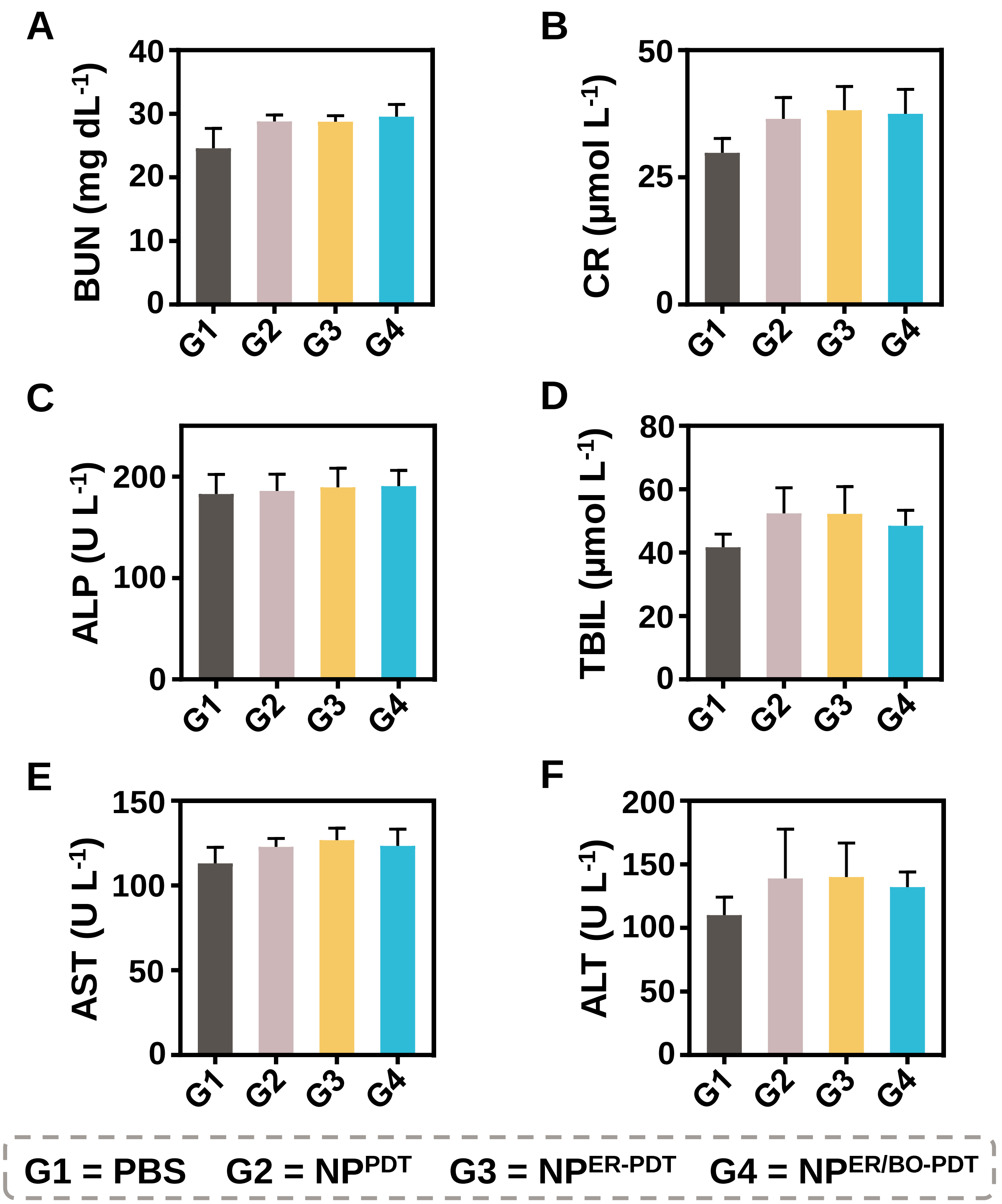

Figure S14. Biosafety evaluation of $NP^{ER/BO-PDT}$ *in vivo*. A-F) Biochemical analysis of serum: A) blood urea nitrogen (BUN); B) serum creatinine (CR); C) alkaline phosphatase (ALP); D) total bilirubin (TBIL); E) aspartate aminotransferase (AST); F) alanine aminotransferase (ALT).

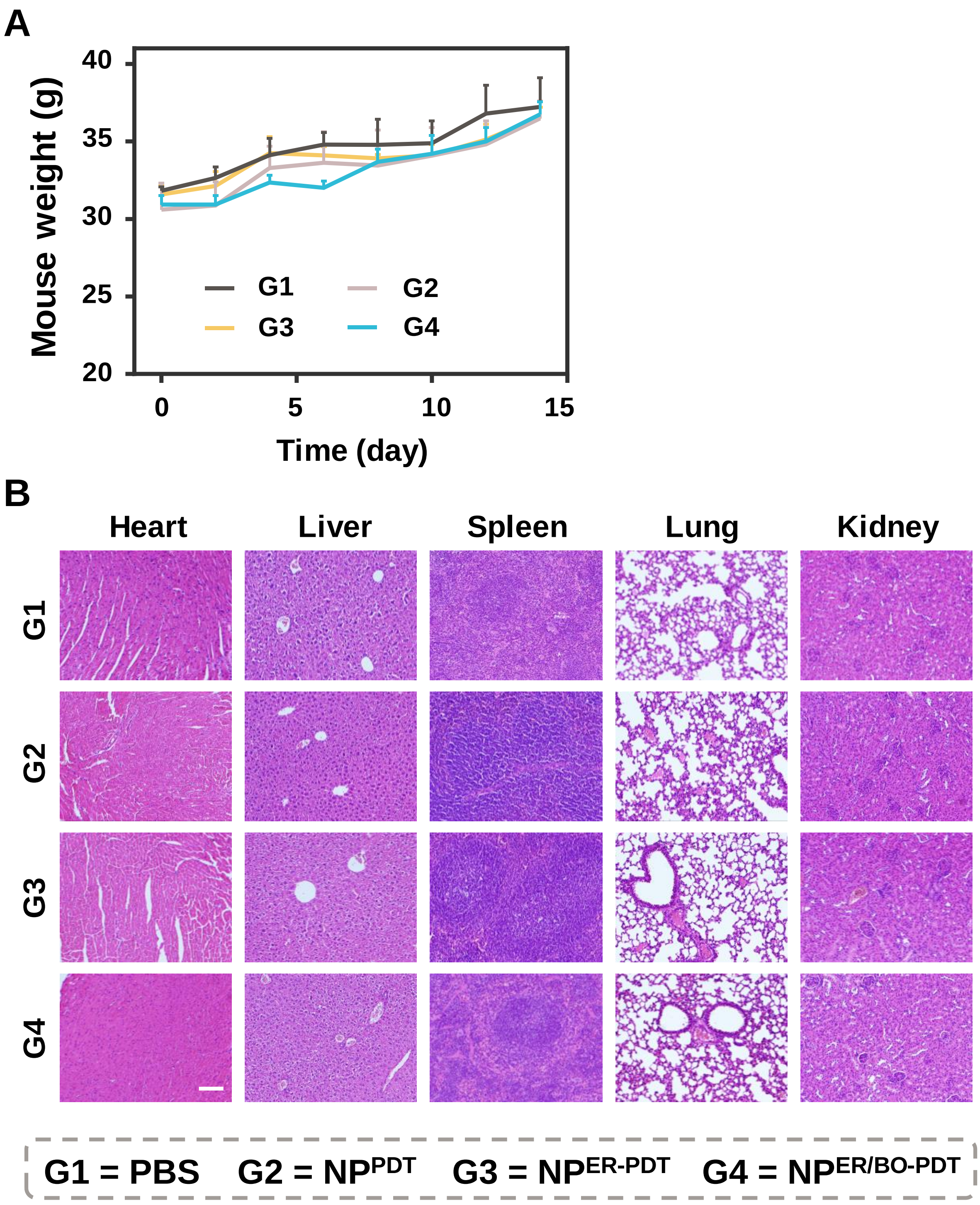

Figure S15. *In vivo* systemic toxicity study. A) Body weight changes of KM mice after various treatments. B) The assessment of main organs function by H&E staining. Scale bar: 100 µm.

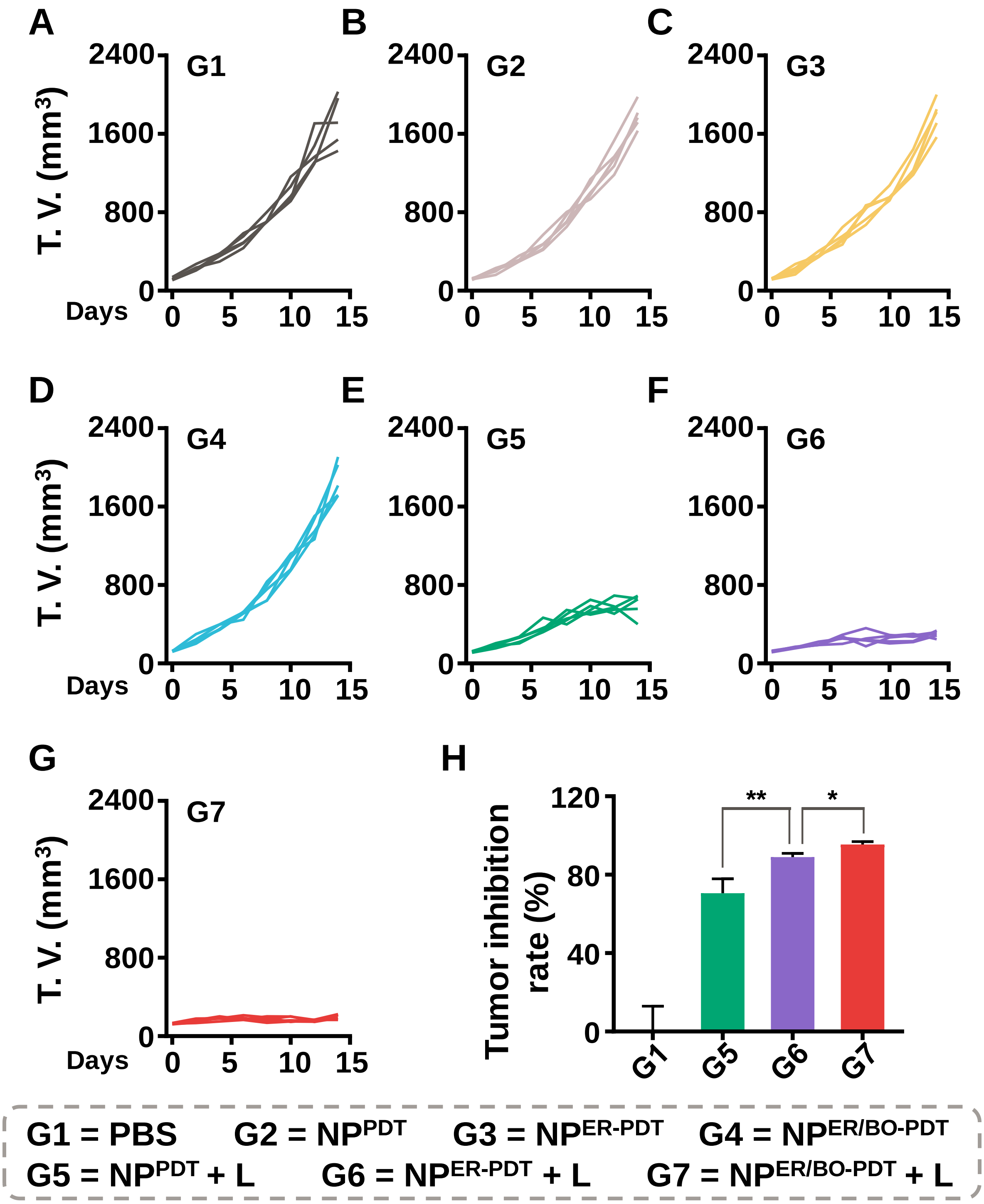

Figure S16. A-G)The individual tumor volume and H) tumor inhibition rate of the orthotopic K7M2 model mice after various treatments. Data are shown as mean ± SD. H) Student's *t*-test. $*p < 0.05$, $**p < 0.01$.

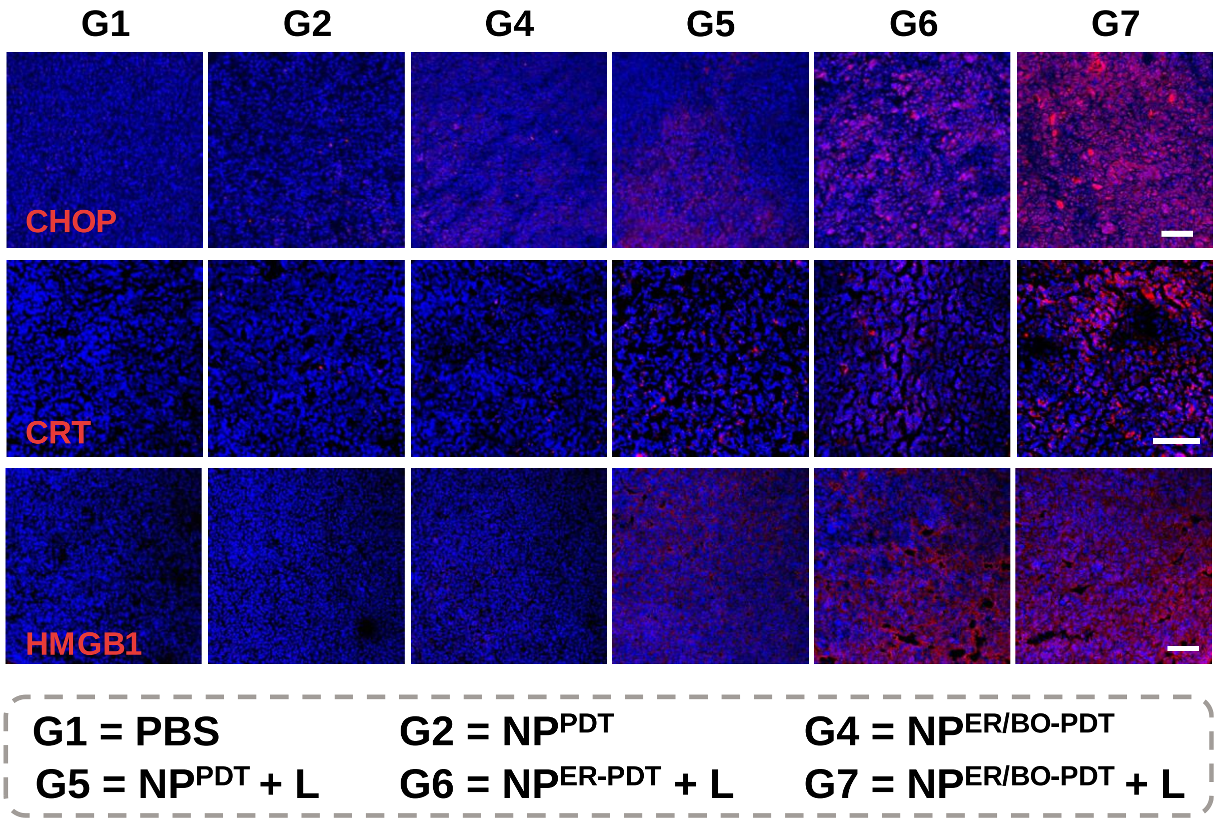

Figure S17. $NP^{ER/BO-PDT}$ induced ER stress and DAMPs release under NIR light irradiation *in vivo*. Immunofluorescence staining of of CHOP (upper), CRT exposure (middle) and HMGB1 release (lower) in an orthotopic K7M2 model mice after various treatments with or without NIR light irradiation. scale bar: 100 μm.

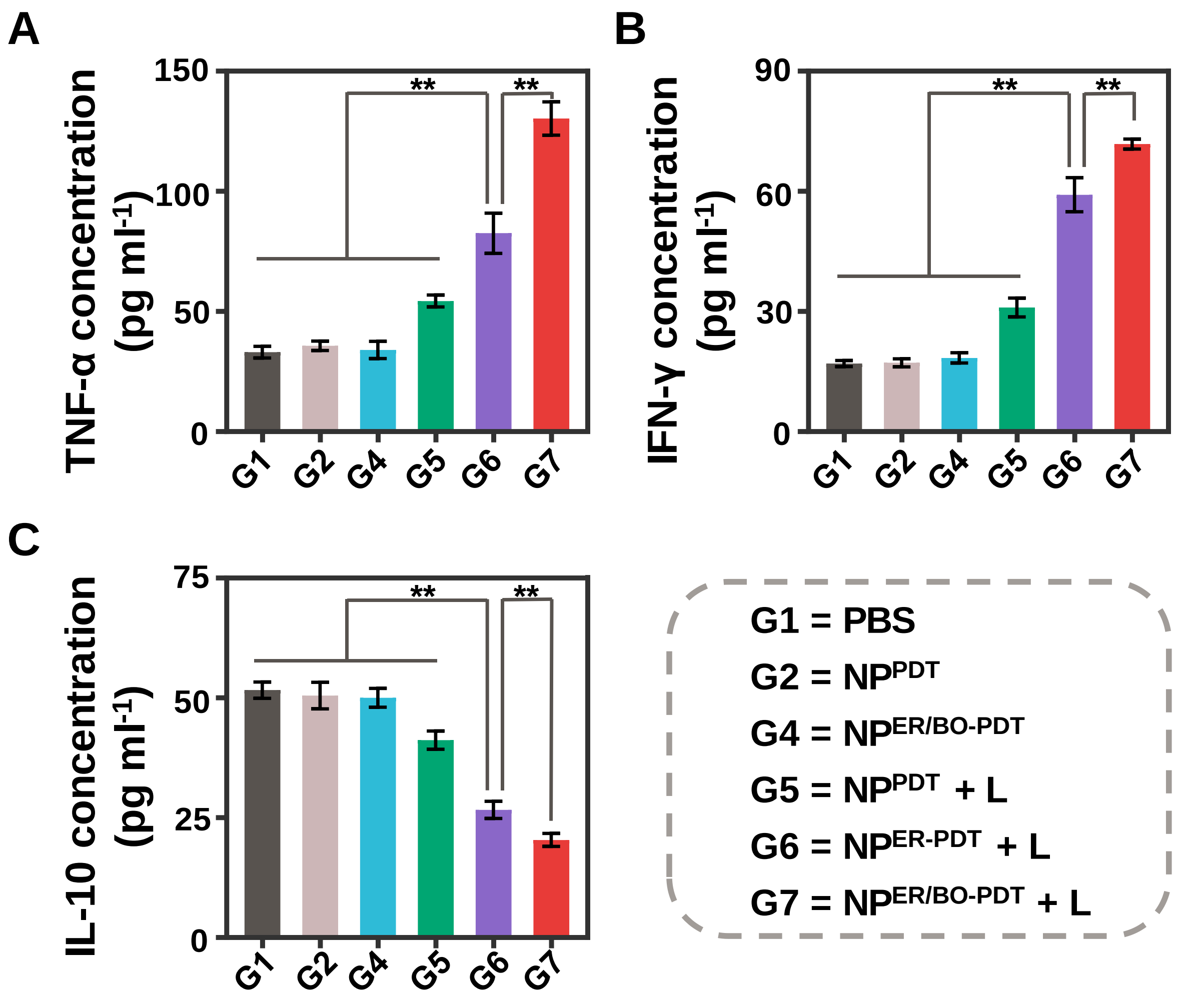

Figure S18. Changes of cell factors about the model mice after various treatments. A) TNF-α, B) IFN- γ and C) IL-10 were detected by ELISA kit. Data are shown as mean ± SD. A-C) Student's *t*-test. ***p* < 0.01.

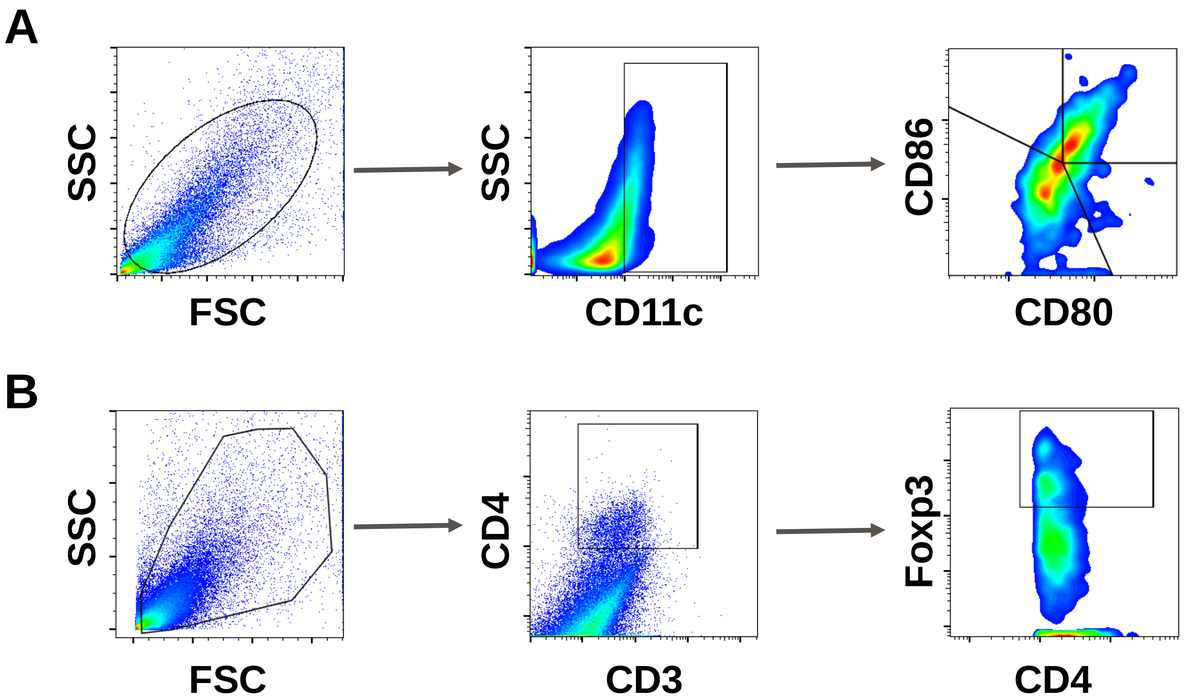

Figure S19. Gating strategies for flow cytometric analysis: A) matured DCs cell (CD11c$^+$CD80$^+$CD86$^+$) within tumor tissues; B) Tregs (CD4$^+$Foxp3$^+$) within tumor tissues.

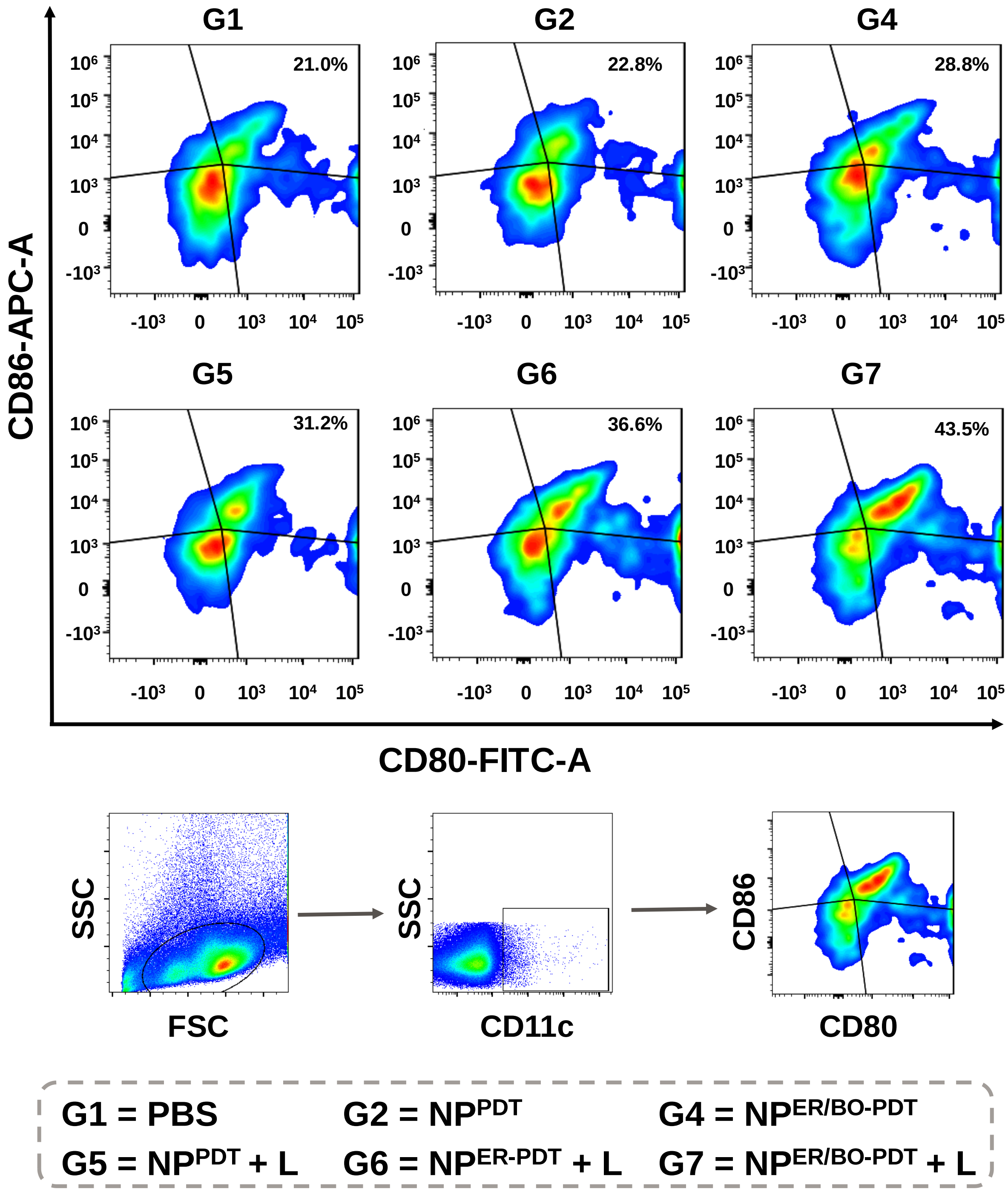

Figure S20. Gating strategies and representative FCM analysis images of matured DCs ($CD11c^{+}CD80^{+}CD86^{+}$) within TDLNs after various treatments.

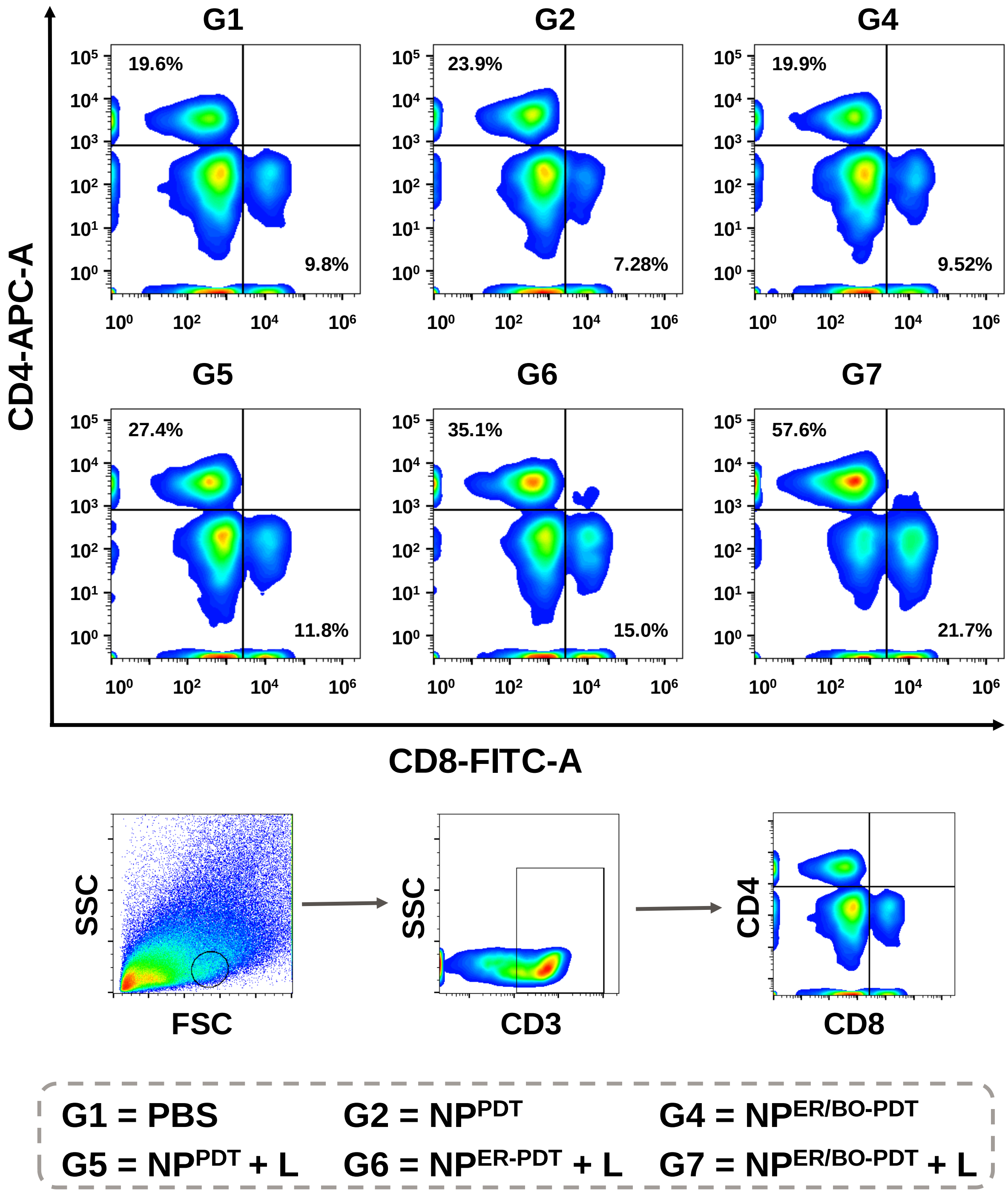

Figure S21. Gating strategies and representative FCM analysis images of T cell ($CD3^+$) within tumor tissues after various treatments.

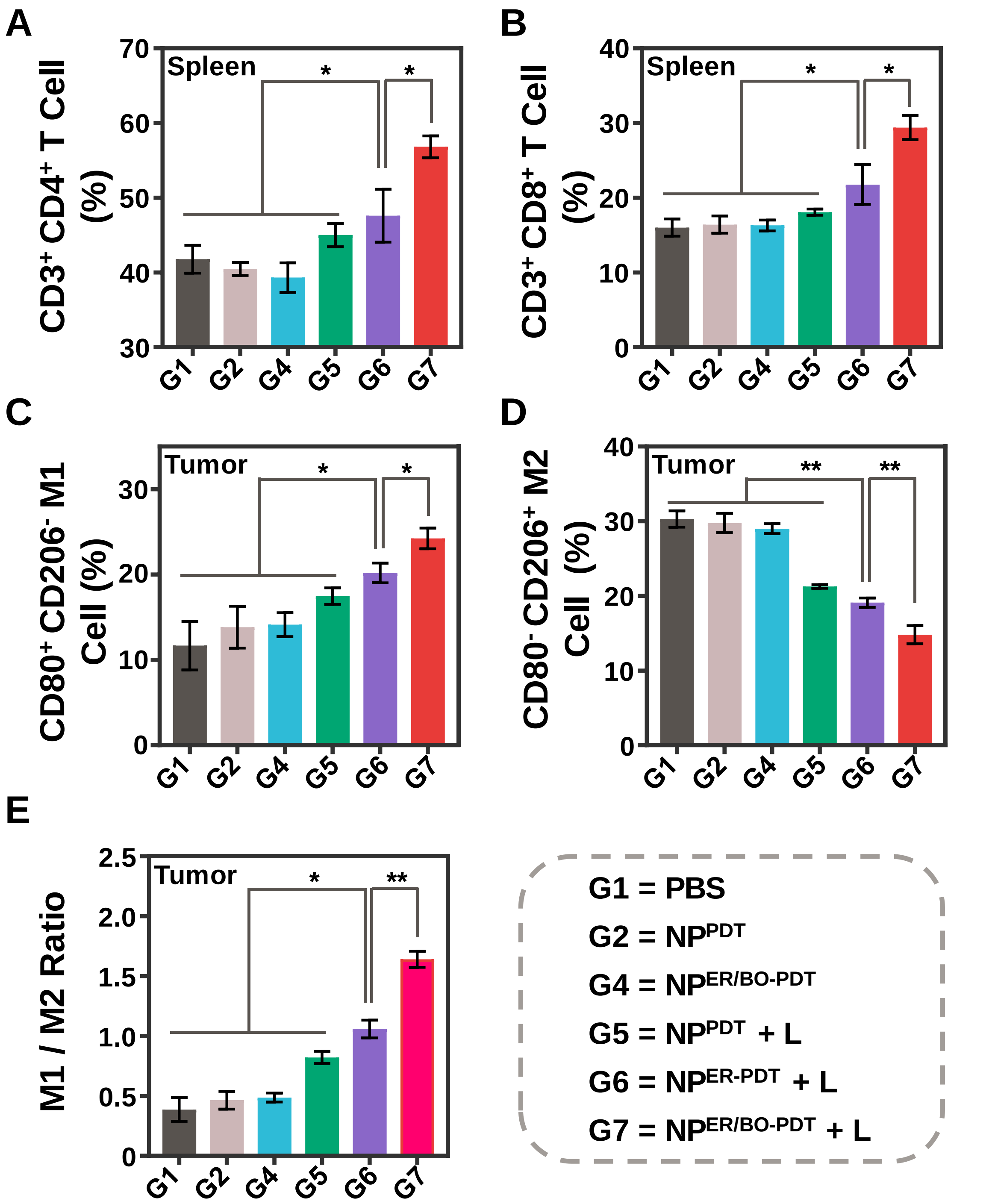

Figure S22. Immune response induced by $NP^{ER/BO\text{-}PDT}$. A) The percentages of population of $CD4^+$ T cells ($CD3^+$ $CD4^+$) and B) $CD8^+$ T cells ($CD3^+$ $CD8^+$) within spleen tissues after various treatments. C) The percentages of population of M1 TAMs ($CD80^+$ $CD206^-$) and D) M2 TAMs ($CD80^-$ $CD206^+$) within tumor tissues after various treatments. E) The rate of M1/M2 within tumor tissues. Data are shown as mean ± SD. A-E) Student's *t*-test. *$p < 0.05$, **$p < 0.01$.

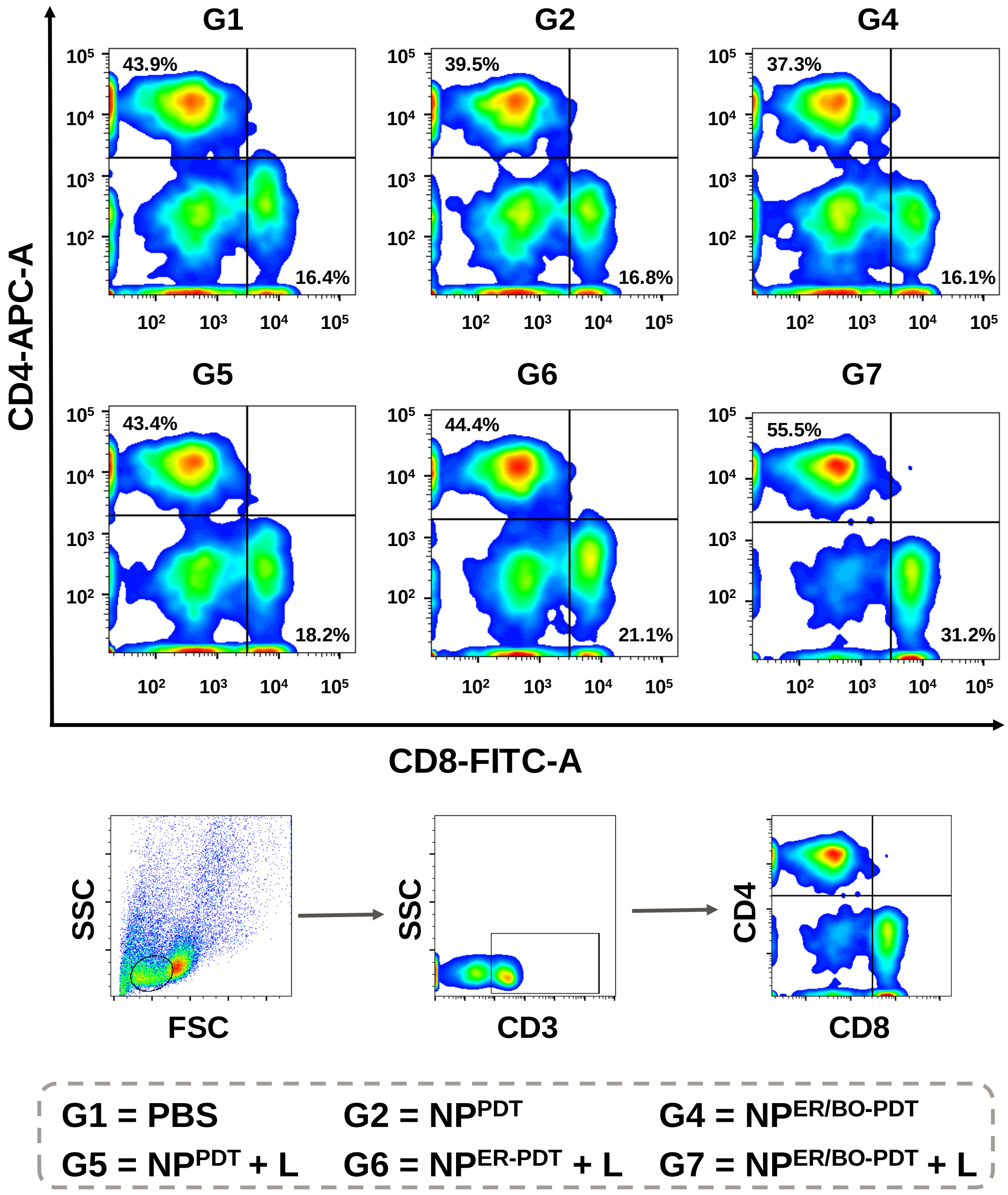

Figure S23. Gating strategies and representative FCM analysis images of T cell ($CD3^+$) within spleen tissues after various treatments.

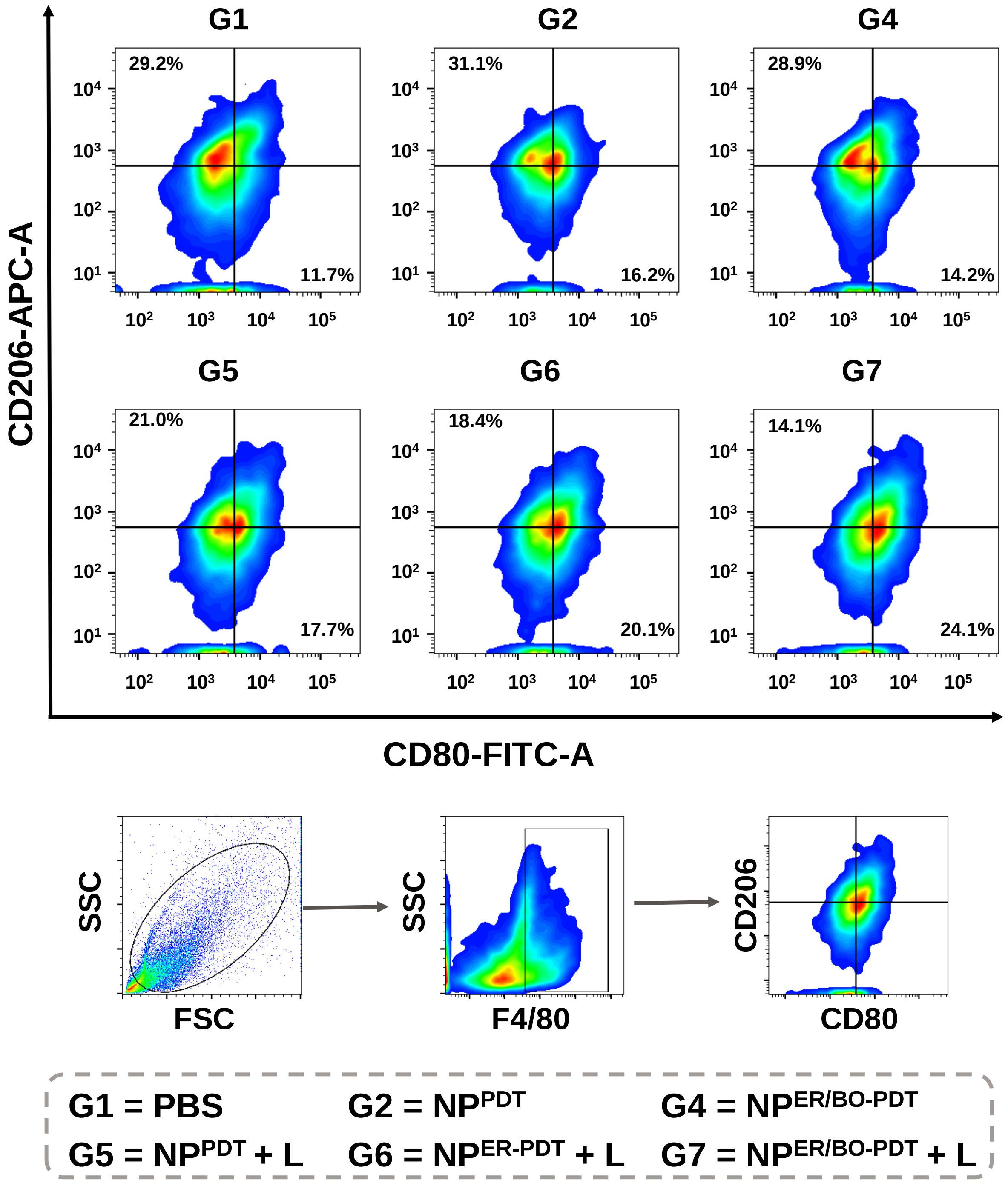

Figure S24. Gating strategies and representative FCM images of M1 macrophages ($CD80^{+}CD206^{-}$) and M2 macrophages ($CD80^{-}CD206^{+}$) within tumor tissues after various treatments.

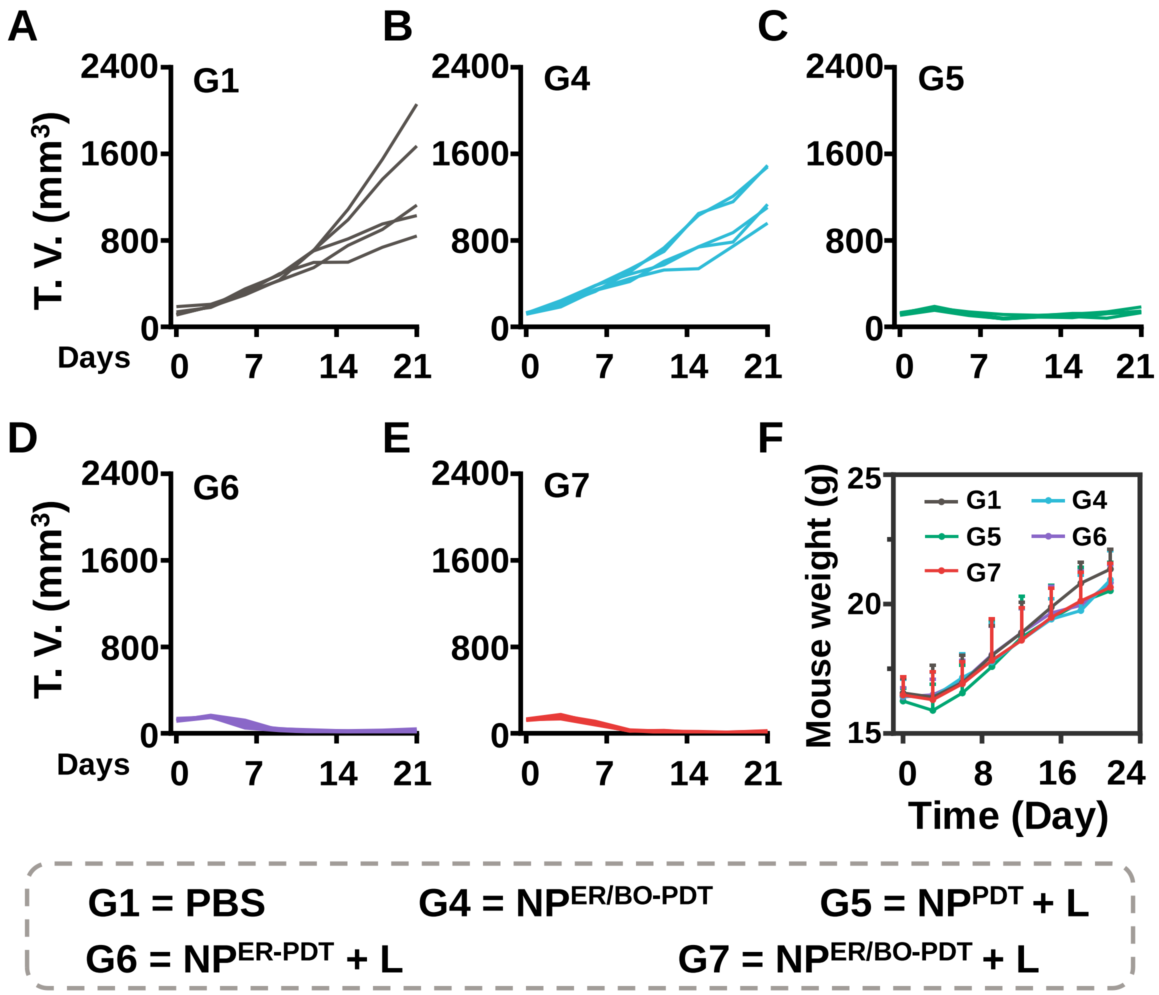

Figure S25. A-E) The individual tumor volume of PDX^{OS} tumor model mice after various treatments. F) The body-weight variation of mice after various treatments.

Table S1. Antibodies used for flow cytometry analysis.

Antibody	Company	Catalog number
Goat polyclonal Secondary Antibody to Rabbit IgG-H&L (Alexa Fluor) ®488	abcam	ab150077
Goat polyclonal Secondary Antibody to Rabbit IgG-H&L (Alexa Fluor) ®555	abcam	ab150078
PE-CD3	elabscience	E-AB-F1013D
APC-CD4	elabscience	E-AB-F1097E
FITC-FOXP3	elabscience	E-AB-F1238C
PC5.5-CD3	elabscience	E-AB-F1013J
FITC-CD8	elabscience	E-AB-F1104C
APC-CD206	elabscience	E-AB-F1161E
PE-F4/80	elabscience	E-AB-F0995D
PE-CD11c	elabscience	E-AB-F0991D
FITC-CD80	elabscience	E-AB-F0992C
APC-CD86	elabscience	E-AB-F1012E

References

[1] D. Wei, Y. Chen, Y. Huang, P. Li, Y. Zhao, X. Zhang, J. Wan, X. Yin, T. Liu, J. Yin, Z. Liu, Q. Zhang, J. Wang, H. Xiao, Nano Today 2021, 41, 101288.

[2] M. Kunitski, N. Eicke, P. Huber, J. Köhler, S. Zeller, J. Voigtsberger, N. Schlott, K. Henrichs, H. Sann, F. Trinter, L.P.H. Schmidt, A. Kalinin, M. S. Schöffler, T. Jahnke, M. Lein, R. Dörner, Nature Communications 2019, 10, 1.
